# Does System Choice Matter? Influence of Hospital Information Systems on Digital Maturity

**DOI:** 10.1007/s10916-026-02450-w

**Published:** 2026-08-08

**Authors:** Jonas Backes, Alexander Geissler, Jonas Subelack, David Ehlig

**Affiliations:** Chair of Health Economics, Policy and Management, School of Medicine, University of St. Gallen, St. Gallen, Switzerland

**Keywords:** Hospital information systems (HIS), Digitalization, Digital maturity, Interoperability

## Abstract

Hospitals worldwide invest heavily in digital infrastructure to improve efficiency, safety, and patient-centeredness. Hospital Information Systems (HIS) form the backbone of these efforts. However, the HIS provider market remains fragmented and heterogeneous, thus potentially influencing hospitals’ ability to achieve digital transformation. Empirical evidence on how HIS choice relates to digital maturity remains limited. Using data from more than 1,600 hospitals participating in the German-wide DigitalRadar (DR) project (i.e., covering about 90% of all hospitals), we examined the relationship between HIS provider characteristics and hospitals’ digital maturity. The DR dataset comprises 234 standardized items per hospital and provides a maturity score from 0 to 100 for each hospital. Four HIS provider features (i.e., module utilization, integration ratio, external provider variation, and maximum hospital size coverage) were derived and used for *k*-means clustering. Multivariate linear regressions, controlling for hospital characteristics, quantified associations with digital maturity. *K*-means clustering identified four clusters of HIS providers. Higher module utilization (β = 6.87, *p* < 0.05) and integration ratios (β = 14.35, *p* < 0.01) were consistently associated with greater digital maturity. External provider variation and maximum hospital size coverage were not significantly associated with overall digital maturity. The choice of HIS provider is associated with hospitals’ digital maturity, particularly with respect to broader module coverage and stronger integration. Providers with low module utilization and limited integration are associated with lower maturity. Our findings highlight the need for strategic HIS procurement, targeted system/organizational improvements, and policy incentives to accelerate digital transformation.

**Supplementary Information:**

The online version contains supplementary material available at 10.1007/s10916-026-02450-w.

## Introduction

Health systems globally have invested substantially in IT infrastructure to achieve safer, more efficient, and more patient-centered care [1, 2]. Programs such as the U.S. HITECH Act, Germany’s Hospital Future Act, the NHS plan for digital health, and Denmark’s National Digital Health Strategy exemplify this transformation toward greater digital maturity [3–6]. In this study, digital maturity refers to the extent to which digitalization is realized in clinical and administrative processes, and the degree to which it is supported by governance structures, organizational capabilities, and resource allocation.

Benefits of greater digital maturity are widely recognized and span the quadruple aim of healthcare delivery developed by Bodenheimer and Sinsky [7]: (i) *improved patient experience* (e.g., patient portals giving real-time access to appointment scheduling, lab results, and discharge summaries), (ii) *enhanced population health* (e.g., automatic dosing recommendations, allergy checks, and drug-drug interaction, reducing diagnostic and medication errors), (iii) *reduced cost per patient* (e.g., elimination of paper-based handoffs enabling faster clinical decisions and shorter length of stay), and (iv) *improved workforce work-life balance* (e.g., single sign-on and automated shift handovers reducing repetitive log-ins and redundant data entry) [8–13].

A central role lies with Hospital Information Systems (HIS), which form the digital backbone of hospital operations. We define HIS as hospital-wide information systems to which a large variety of modules (e.g., Radiology Information Systems [RIS], Laboratory Information Systems [LIS]) are connected [14]. Numerous interfaces connect the HIS with its modules, ensuring central data availability across departments [15]. Today, the HIS market is fragmented, with providers offering heterogeneous systems. Consequently, hospitals often face *technical*, *operational*, and *strategic* barriers when implementing and operating an HIS.

*Technically*, the lack of interoperability standards often necessitates custom interfaces, leading to fragmented systems, data silos, and media discontinuities [16]. HIS built on and operated using outdated programming languages remain common [17]. Unsurprisingly, as of 2024, in Germany no hospital yet operated fully cloud-based [18]. *Operationally*, HIS platforms historically prioritized billing over clinical functionality, frequently misaligning with clinicians’ workflows and prompting workarounds that interrupt care and compromise efficiency and safety [19, 20]. *Strategically*, provider lock-in constrains agility regarding module upgrades, pricing, and third-party integration. We recognize that new standards (e.g., Fast Healthcare Interoperability Resources [FHIR]) aim to address some of these challenges [21]. However, these issues remain prominent today. This heterogeneity suggests that the choice of an HIS is related to the extent to which hospitals can achieve digital transformation.

Germany offers an informative setting to study the role of HIS providers in digital transformation. First, the country operates one of Europe’s largest hospital systems (~ 1,700 acute-care and psychiatric hospitals) with 16 federal states financing hospital infrastructure, while operating costs are reimbursed mainly through statutory health insurance [22]. Following the Hospital Future Act of 2020, the German Ministry of Health commissioned the DigitalRadar (DR) consortium to develop and monitor nationwide digital maturity. Because participation is a precondition for hospitals to receive funding, the DR yields standardized maturity data for ~ 90% of hospitals, a coverage level rarely available internationally [23, 24]. Second, Germany remains at an inflection point. Years of underinvestment resulted in hospitals lagging behind international peers in digital maturity [25]. Functions such as electronic prescribing first became mandatory in 2024, while the roll-out of electronic health records started in 2025, both long after the Nordic and Baltic countries [26]. Yet, according to the European Commission’s 2024 Digital Decade eHealth assessment [27], Germany recently showed one of the EU’s largest annual improvements in the digital availability of health data to patients. Third, the German HIS market facilitates provider-level analysis. With more than 20 active providers, the market offers sufficient variation to identify characteristics associated with digital maturity. The absence of a nationally mandated HIS standard leaves procurement decisions to individual hospitals, allowing provider choice to influence digitalization pathways. Furthermore, Hospital Future Act funds are flowing through HIS providers, making it particularly important to understand which provider characteristics are associated with higher digital maturity.

Although previous studies have examined user acceptance [28], profitability [13], and success factors in hospital digitalization [29], gaps remain. Currently, there is no scientific evidence on whether the choice of the HIS affects a hospital’s digital maturity. This led us to the following research questions:

1. Which features of an HIS (i.e., module utilization, integration ratio, external provider variation, and maximum hospital size coverage) are most strongly associated with hospitals’ digital maturity?
2. To what extent is the HIS provider choice related to hospitals’ digital maturity?

Results are relevant for hospitals making informed IT procurement choices, HIS providers seeking to align system and portfolio design, and policymakers targeting digitization incentives.

Using a nationwide survey from the German DigitalRadar (DR) in 2021 [24], covering  ~ 90% of all hospitals in Germany, we analyzed the digital maturity of more than 1,600 hospitals. We extracted hospital-level data on digital maturity, the deployed HIS providers and IT modules, and hospital characteristics. We developed four features to characterize HIS providers. Providers were clustered via *k*-means and validated through silhouette scores and bootstrap analysis. Multivariate linear regressions quantified the relationships between HIS provider characteristics and hospitals’ digital maturity.

## Methods

### Data Sourcing from a Nationwide Survey and Cleaning

Data originate from the German DR project, a nationwide survey consisting of 234 questions per hospital, from which we extracted three types of data: *DR-scores* (one overall digital maturity score, seven dimensions, and four workflow-related scores), *HIS details*, and *hospital characteristics*.

*DR-scores* are expressed as an overall score and range from 0 (not digitalized) to 100 (fully digitalized). The score was developed by a national consortium and is based on a scoping review, stakeholder input, and pilot testing [24]. The overall DR maturity can be disaggregated into seven dimension scores and four workflow scores (Supplementary Material 1).

*HIS data* included the main provider, presence and vendor of 17 modules, and the integration status of modules (integrated, non-integrated, or part of the HIS suite*)*. *Hospital characteristics* comprised the hospital type (teaching, university, psychiatric), ownership (public, private non-profit, private for-profit), capacity (number of beds), HIS provider module share (percentage of modules supplied by HIS provider), and location (federal state). Additionally, we controlled for hospital chain affiliation, as chains often share IT systems, standards, strategies, and procurement processes across sites, affecting digital maturity. The exact methodology for the maturity score calculation is provided by Geissler et al. [24].

The DR survey was completed by multidisciplinary hospital teams (typically consisting of IT, clinical, and administrative staff) via an internal self-assessment coordinated by a designated hospital lead. The DR project consortium provided guidance and support throughout the process (e.g., webinars and help desks) and applied centralized auditing checks before issuing *DR-scores* [24]. The author team conducted additional cleaning including harmonization of provider and module names, exclusion of incomplete records, and validation of information against hospital websites.

### Creation of HIS Features

We constructed four features to characterize HIS providers (Table 1). We selected these features because they capture structural characteristics relevant to hospitals’ digital transformation: breadth of in-house functionality, openness to external systems, diversity of external connectivity, and scalability across hospital complexity levels. Additionally, due to confidentiality constraints that required anonymizing HIS providers, provider-level characteristics were used to enable comparisons beyond vendor identities. All four features are constructed from hospital-level data and aggregated to the HIS provider level.

**Table 1:** Definition of features used to characterize HIS providers

Variable	Name	Description
$\:{V}_{1,p}$	Module Utilization	Ratio of distinct modules offered by HIS provider *p* to the total of 17 possible modules
$\:{V}_{2,p}$	Integration Ratio	Ratio of connected external modules to total external modules across hospitals using HIS provider *p*
$\:{V}_{3,p}$	External Provider Variation	Count of distinct external module providers that are integrated with HIS provider *p*
$\:{V}_{4,p}$	Max. Hospital Size Coverage	Maximum number of beds among hospitals using HIS provider *p*

Features are defined at the HIS provider level *p* and are based on data from the DigitalRadar survey 2021

(i) Module Utilization

Measures the breadth of modules offered by the HIS provider * p* relative to the total number of possible modules (here, 17; please see module long-list in Supplementary Material 2). The ratio ranges from 0 (no modules offered) to 1 (all possible modules offered) and can be displayed as:

$$V_{1,p} = \sum_{J=1}^{17} 1_{p,j} / 17$$

$\:{1}_{p,j}$ is defined as a binary variable with 1 if at least one hospital using HIS provider *p* has also module *j*, and 0 otherwise. The different modules are indexed with j $\:\in\:\:\left\{1,\:\dots\:,\:17\right\}$. This ratio reflects the architectural opportunity provided by the HIS, with high values indicating the opportunity for hospitals to build a monolithic architecture (i.e., single provider offers a broad suite of modules), whereas low values may constrain hospitals toward a best-of-breed architecture (i.e., specialized systems from various providers) [30]. Haas and Kuhn [31] outline that hospitals deploying monolithic architectures benefit from fewer interfaces, standardized data structures, less maintenance, and tighter integration across departments. We therefore hypothesize that higher utilization is positively associated with digital maturity.

(ii) Integration Ratio

Assesses the degree to which external modules (i.e., modules sourced from vendors other than the HIS provider) are integrated with the core HIS environment. The ratio ranges from 0 (no external modules connected) to 1 (all external modules connected) and can be displayed as:

$$V_{2,p} = \sum\limits_{h \in H_p} c_h \Big/ \sum\limits_{h \in H_p} e_h \quad , \text{with } V_{2,p} = 0 \quad \text{if } \sum\limits_{h \in H_p} e_h = 0$$

where $\:{H}_{p}$ is the set of all hospitals using provider *p*. For each hospital $\:h\in\:{H}_{p}$: $\:{e}_{h}$ denotes the total number of external modules present in hospital * h*, and $\:{c}_{h}$ the number of those external modules that are integrated with the core HIS. Following the DR classification [24], modules are considered external if sourced by a vendor other than the HIS provider * p* and integrated if technically connected via an interface. V₂,ₚ therefore represents the share of external modules across all hospitals using provider * p* that are technically connected to the core HIS. If $\:{\sum\:}_{h\in\:{H}_{p}}{e}_{h}=0$, no external modules are present. The ratio indicates openness to external modules and serves as a proxy for the vendor’s interoperability and ability to exchange information with external systems. Lehne et al. [32] argue that fragmented systems hinder large-scale data processing and efficient information retrieval, and impede reductions in documentation burden and patient participation. Hence, we hypothesize that a higher ratio is positively associated with digital maturity.

(iii) External Provider Variation

Captures the diversity of external module providers that are integrated with HIS provider *p*. It is defined as the count of unique external providers whose systems are integrated with the HIS and can be displayed as:

$$V_{3,p} = \bigcup\limits_{h \in H_p} Q_h$$

which represents the union of all external modules that have at least one connected system in hospital *h* using HIS *p*. It shows the diversity of integrated external vendors of modules and serves as a proxy for enhanced flexibility when connecting with external systems. Zhu and Cennamo [33] argue that more open systems reduce vendor lock-in and allow users to complement their core systems with best-in-class external systems, closing capability gaps of the main vendor’s portfolio. We therefore hypothesize that a higher variation is positively associated with digital maturity.

(iv) Maximum Hospital Size Coverage

Reflects the largest hospital size (bed count) supported by HIS provider *p*. While ranges are reported to avoid re-identification of providers, analyses used continuous bed counts. With this variable we account for providers’ ability to offer their product to larger hospitals and thus handle complex workflows. Haas and Kuhn [31] note that larger hospitals impose different requirements than their smaller counterparts. Based on Levitt and March’s theory of organizational learning [34], providers with HIS installed at large hospitals have built capabilities that remain embedded and benefit their wider installed base. We therefore hypothesize that a higher coverage is positively associated with digital maturity.

### Identification of HIS Clusters

We applied *k*-means clustering to group HIS providers into clusters of similar features, enabling comparison beyond individual providers and revealing structural patterns that provider-level analyses would obscure [35]. Each HIS provider was treated as one observation, represented by its vector ($\:{V}_{1,p}$ to $\:{V}_{4,p}$ in our base model; $\:{V}_{1,p}$ to $\:{V}_{5,p}$ in our alternative model). All features were standardized with z-scores. HIS providers were iteratively assigned to their respective clusters by minimizing squared Euclidean distance to cluster centers (i.e., centroids) until convergence [36].

Several methods exist to identify the optimal number of clusters *k* [35]. We used two complementary metrics: First, the “elbow” method based on the within-cluster sum of squares (WSS), with the optimal *k* being defined as the inflection point beyond which additional clusters yield only marginal reductions in WSS. Second, the average silhouette score to assess cluster differentiation. Initialization bias was reduced by selecting 50 random starting points for cluster creation and selecting the solution with the lowest variance in WSS and silhouette values. We set a minimum threshold of three providers per cluster to avoid overly small and unstable clusters. We performed a bootstrapping stability analysis (100 resamples) to verify the reproducibility of clusters. Stability was expressed by the Jaccard Index (range 0–1), with 1 indicating perfect stability. Lastly, centroids were examined to identify the distinguishing features of each HIS cluster. Final clusters form the basis for grouping HIS providers for statistical modelling. Clusters were stratified by hospital size, ownership structure, teaching type, and federal state to describe the HIS landscape.

### Statistical Modelling

We first applied a simple Ordinary Least Squares (OLS) regression to test whether hospitals using specific HIS providers differed in digital maturity by regressing provider identifiers on the total DR-score. Next, we applied multivariate linear regression with DR-score as the primary dependent variable and DR-score dimensions and workflow scores as secondary outcomes. The four HIS features act as independent predictors. To account for structural heterogeneity, we included hospital-level variables: the number of beds, ownership type, hospital type, IT full-time equivalent (FTE) per bed, share of modules covered by HIS provider, hospital chain, and federal state. Hospital type was categorized as teaching, university, or psychiatric. Teaching hospitals provide clinical training but are not directly affiliated with a university/medical faculty. The following OLS regression is used to assess the relationships between the HIS features and digital maturity:

$$\:{DRS}_{i}=\:{\beta\:}_{0}+\:\sum\:_{j=1}^{4}{\beta\:}_{j}*\:{HIS\_Feature}_{ij}+\:{C}_{i}^{{\prime\:}}\gamma\:+{\epsilon\:}_{i}$$

where $\:{DRS}_{i}$ denotes the digital maturity score of hospital * i*, using either the total, dimension, or workflow scores. $\:{HIS\_Feature}_{ij}$ represents the value of HIS feature * j* for hospital * i*. $\:{C}_{i}$ is the vector of hospital-level control variables (e.g., number of beds) with the corresponding coefficient vector $\:\gamma\:$. The error term is shown by $\:\epsilon\:$. Finally, we ran OLS regression with HIS provider clusters to gain further insights into the associations between cluster assignment and DR-scores.

OLS diagnostics included checks of normality of residual distributions via Quantile-Quantile (Q-Q) plots and histograms, multicollinearity among hospital-level controls using Variance Inflation Factors (VIF), and heteroscedasticity. Skewness and outliers were addressed through transformation and winsorization at the 2.5th and 97.5th percentiles. Additionally, to address multiple testing concerns across secondary outcomes, we applied Benjamini-Hochberg false discovery rate (FDR) correction per predictor at the threshold of q = 0.05 [37].

As a sensitivity analysis, we constructed an additional fifth feature, *Cloud Offering* ($\:{V}_{5,p}$), indicating whether HIS provider *p* offers a cloud-based solution in its portfolio. We re-ran all clustering and multivariate regressions with this variable. Additionally, we re-estimated regressions excluding the *Structures and Systems* dimension to assess potential overlap with HIS feature inputs (i.e., module availability and integration status). All other DR dimensions and workflow scores are independent of the HIS feature construction. We performed all analyses in R version 4.5.0.

## Results

### General Data Description

The dataset included 1,607 hospitals, after excluding 17 hospitals that did not specify their HIS. Regarding hospital size, 56.2% of the hospitals included had fewer than 250 beds. Public ownership was most frequent (37.6%) (Table 2). The mean DR-score was 38.24 (SD: 10.83). Across DR-score dimensions, maturity was highest for *Structures and Systems* (mean: 55.71; SD: 17.26) and lowest for *Patient Participation* (mean: 5.25; SD: 8.95). Among clinical workflows, *Administrative* processes reached the highest maturity (mean: 43.96; SD: 13.49), whereas *Discharge* management was the least mature (mean: 11.31; SD: 10.02). Hospitals reported 24 different HIS providers, including one category for self-developed HIS. The three most frequently deployed providers cover 66% of hospitals. The 17 modules were supplied by 112 distinct vendors. The number of running licenses per module is shown in Supplementary Material 2. Lastly, 52% of the modules present at a hospital were on average supplied by its HIS provider (SD: 0.26).

**Table 2:** Descriptive statistics of identified HIS provider clusters

		HIS provider clusters			
	Total (*N* = 1,607)	Cluster A (*N* = 1,301)	Cluster B (*N* = 217)	Cluster C (*N* = 7)	Cluster D (*N* = 82)
**DR-score**					
Mean (SD)	38.24 (10.83)	38.55 (10.36)	40.68 (11.38)	21.76 (8.14)	28.14 (10.3)
**DR-score dimension– Mean (SD)**					
Structures & Systems	55.71 (17.26)	56.22 (16.33)	60.76 (17.4)	24.53 (10.8)	36.83 (17.11)
Resilience Mgmt. & Performance	45.38 (15.76)	45.24 (15.69)	48.23 (15.99)	40.32 (11.85)	40.36 (15.08)
Organizational Control & Data Mgmt.	40.64 (13.31)	40.61 (13.07)	44.8 (13.14)	20.88 (11.11)	31.89 (11.83)
Clinical Processes	38.76 (13.93)	39.58 (13.31)	39.82 (14.31)	15.09 (8.75)	24.87 (13.99)
Information Exchange	25.18 (10.15)	25.53 (9.91)	26.18 (10.7)	12.92 (8.52)	17.99 (9.28)
Telehealth	18.1 (14.03)	18.35 (14.12)	19.2 (12.74)	15.34 (17.74)	11.44 (13.99)
Patient Participation	5.25 (8.95)	4.84 (8.09)	8.29 (13.01)	6.56 (14.78)	3.46 (6.25)
**DR Workflow-scores – Mean (SD)**					
Administrative	43.96 (13.49)	43.97 (13.39)	47.14 (13.35)	31.38 (9.03)	36.52 (12.3)
Admission	22.27 (11.63)	22.56 (11.34)	24.06 (11.85)	16.07 (15.05)	13.57 (11.69)
Inpatient Treatment	34.17 (12.29)	34.83 (11.73)	35.18 (13.07)	13.86 (7.02)	22.81 (12.32)
Discharge	11.31 (10.02)	11.06 (9.45)	14.19 (12.68)	8.05 (13.18)	8.06 (8.92)
**Hospital type (%)**					
Teaching	878 (54.64)	727 (55.88)	135 (62.21)	1 (14.29)	15 (18.29)
University	54 (3.36)	31 (2.38)	22 (10.14)	0 (0.00)	1 (1.22)
Psychiatric	251 (15.62)	208 (15.99)	13 (5.99)	5 (71.43)	25 (30.49)
**Ownership status (%)**					
Public	604 (37.58)	530 (40.74)	42 (19.35)	3 (42.86)	29 (35.37)
Private non-profit	456 (28.38)	332 (25.52)	78 (35.94)	3 (42.86)	43 (52.44)
Private for-profit	547 (34.04)	439 (33.74)	97 (44.70)	1 (14.29)	10 (12.20)
**Number of beds**					
Mean (SD)	311.28 (315.77)	300.58 (270.99)	462.41(501.43)	48.86 (59.75)	103.51 (122.68)
< 250 beds (%)	903 (56.20)	720 (55.34)	103 (47.47)	7 (100.00)	73 (89.02)
250 to 500 beds (%)	416 (25.90)	354 (27.21)	54 (24.88)	0 (0.00)	8 (9.76)
501 to 700 beds (%)	141 (8.80)	123 (9.45)	17 (7.83)	0 (0.00)	0 (0.00)
> 700 beds (%)	147 (9.10)	104 (7.99)	43 (19.82)	0 (0.00)	1 (1.22)
**IT FTE per hospital bed**					
Mean (SD)^1^	0.02 (0.03)	0.02 (0.03)	0.03 (0.02)	0.03 (0.03)	0.04 (0.05)
**HIS provider module share**					
Mean (SD)	0.52 (0.26)	0.56 (0.24)	0.35 (0.23)	0.38 (0.49)	0.36 (0.35)
**Federal state (%)**					
Baden-Württemberg	167 (10.39)	127 (9.76)	28 (12.90)	0 (0.00)	12 (14.63)
Bavaria	308 (19.17)	241 (18.52)	34 (15.67)	1 (14.29)	32 (39.02)
Berlin	54 (3.36)	43 (3.31)	8 (3.69)	0 (0.00)	3 (3.66)
Brandenburg	54 (3.36)	39 (3.00)	14 (6.45)	0 (0.00)	1 (1.22)
Bremen	12 (0.75)	10 (0.77)	2 (0.92)	0 (0.00)	0 (0.00)
Hamburg	33 (2.05)	22 (1.69)	5 (2.30)	0 (0.00)	6 (7.32)
Hesse	124 (7.72)	110 (8.46)	12 (5.53)	1 (14.29)	1 (1.22)
Lower Saxony	36 (2.24)	30 (2.31)	6 (2.76)	0 (0.00)	0 (0.00)
Mecklenburg-Western Pomerania	160 (9.96)	128 (9.84)	25 (11.52)	1 (14.29)	6 (7.32)
North Rhine-Westphalia	324 (20.16)	293 (22.52)	23 (10.60)	0 (0.00)	8 (9.76)
Rhineland-Palatinate	82 (5.10)	69 (5.30)	11 (5.07)	0 (0.00)	2 (2.44)
Saarland	19 (1.18)	13 (1.00)	5 (2.30)	1 (14.29)	0 (0.00)
Saxony	79 (4.92)	55 (4.23)	21 (9.68)	0 (0.00)	3 (3.66)
Saxony-Anhalt	49 (3.05)	38 (2.92)	10 (4.61)	0 (0.00)	1 (1.22)
Schleswig-Holstein	64 (3.98)	49 (3.77)	5 (2.30)	3 (42.86)	7 (8.54)
Thuringia	42 (2.61)	34 (2.61)	8 (3.69)	0 (0.00)	0 (0.00)
**Hospital chain (%)** ^2^					
Chain-affiliated	987 (61.42)	830 (63.80)	124 (57.14)	2 (28.57)	31 (37.80)
Individual hospitals	620 (38.58)	471 (36.20)	93 (42.86)	5 (71.43)	51 (62.20)

Values are mean (SD) for continuous variables and frequency (%) for categorical variables. The total column summarizes overall hospital characteristics across all four HIS provider clusters. Percentages refer to the proportion of hospitals within each HIS provider cluster. Only the three federal states with the highest number of hospitals are displayed. (1) Log-transformed and winsorized at 2.5th and 97.5th percentiles for regression analyses to correct skewness and outliers. (2) For descriptive analyses, hospital chain affiliation is reported as a binary variable (chain-affiliated vs. individual hospital). In the regression analyses, hospital chain is included as a categorical variable

### Taxonomy of HIS Providers

Providers averaged a *Module Utilization* of 0.43, indicating ~ 7.5 integrated modules per provider. Notably, three providers use no modules, while one achieves full utilization. The mean *Integration Ratio* was 0.60, ranging from 0 to 1. External connectivity varied widely: five providers integrated with > 50 distinct vendors, with a mean of 20. *Maximum Hospital Size Coverage* ranges from ~ 10 to > 3,000 beds, with only 8 providers active in hospitals with more than 700 beds and 3 in hospitals with more than 2,000 beds. Detailed results per HIS variable and anonymized HIS provider are provided in Supplementary Material 3.

**Fig. 1:**
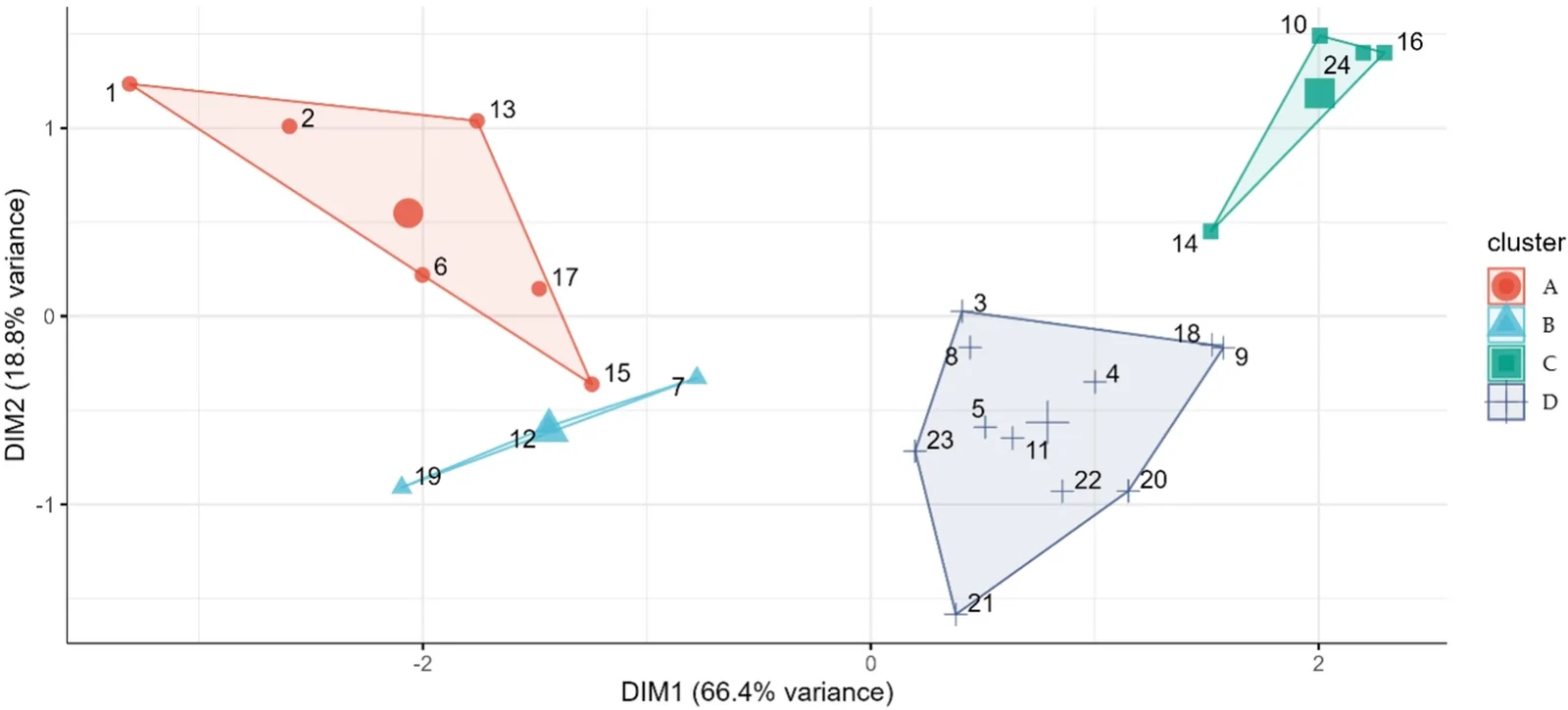
Clustering graph for k = 4 clusters based on HIS variable V1 to V4 Note: Results of *k*-means clustering are shown in a reduced two-dimensional space. Axes correspond to weighted combinations of HIS features that best summarize the information in two-dimensional space. Each point represents a single HIS provider, colored by membership in the respective cluster. Lines are drawn around the provider cluster to illustrate boundaries. Large shapes within each cluster show centers

We determined the optimal number of clusters *k* by plotting the WSS and average silhouette score across *k* = 2 to 10 (Supplementary Material 4–6). The WSS elbow plot showed a steep decline until *k* = 4. WSS reductions were − 50.0, − 14.8, − 9.8, and − 4.1 for *k* = 2 to 5, more than halving between *k* = 4 and 5 and plateauing thereafter. The silhouette score was highest at *k* = 4 (0.476; tied with *k* = 2), exceeding *k* = 3 (0.454) and *k* = 5 (0.398) (Supplementary Material 5). Unlike *k* = 2, the four-cluster solution preserved meaningful provider heterogeneity, satisfied the minimum cluster size criterion, and showed acceptable bootstrap stability. Hence, *k* = 4 was selected. Figure 1 visualizes the *k*-means clustering results. Table 3 presents the centroids defining the four distinct HIS provider clusters:

**Table 3:** Overview of cluster centroids per HIS variable and descriptions

		Cluster centroids					
		$\:{V}_{1,p}$	$\:{V}_{2,p}$	$\:{V}_{3,p}$	$\:{V}_{4,p}$		
Cluster	**Number of** **HIS provider**	Module Utilization	Integration Ratio	External Provider Variation	Hospital Size Coverage	**Jaccard ** **index**	**Description**
Cluster A	6	0.79	0.74	69.83	1,513	0.79	High module utilization and integration ratio, high integration with external IT systems, and deployed in large hospitals
Cluster B	3	0.86	0.78	3.33	1,903	0.57	Very high internal capability and scalability, but limited external system diversity, also used in large hospital environments
Cluster C	4	0.10	0.08	0.25	69	0.68	Very low capability across all dimensions, minimal external connectivity, deployed in very small hospitals
Cluster D	11	0.22	0.68	5.91	250	0.77	Moderate integration ratio with low-to-moderate module utilization, used in smaller to mid-sized hospitals

Each row summarizes one of the four identified HIS provider clusters. To identify key characteristic cluster centroids within each HIS variable column can be compared. The description summarizes the structural differences between provider clusters. HIS provider names are not reported due to confidentiality constraints; the provider-cluster mapping is available upon reasonable request

- Cluster A: high module utilization and integration, typically deployed in large hospitals (*N* = 6 HIS providers).
- Cluster B: high module utilization but limited external connectivity (*N* = 3).
- Cluster C: very low utilization and integration ratio, predominantly serving small or psychiatric hospitals (*N* = 4).
- Cluster D: moderate module utilization and integration ratio, mainly in small- to mid-sized hospitals (*N* = 11).

Cluster sizes ranged from 1,301 hospitals (Cluster A) to 7 hospitals (Cluster C) (Table 2). Given the small size of Cluster C (*N* = 7), regression results for this cluster require cautious interpretation. Cluster B consistently shows the highest digital maturity, particularly in the *Structures and Systems* domain and *Administrative* workflows. Conversely, Cluster D has lower maturity scores, especially for *Telehealth*. Both Clusters C and D exhibit low digital maturity in *Discharge* workflows. Finally, Clusters A and B are concentrated among larger hospitals.

### HIS Market Landscape

**Fig. 2:**
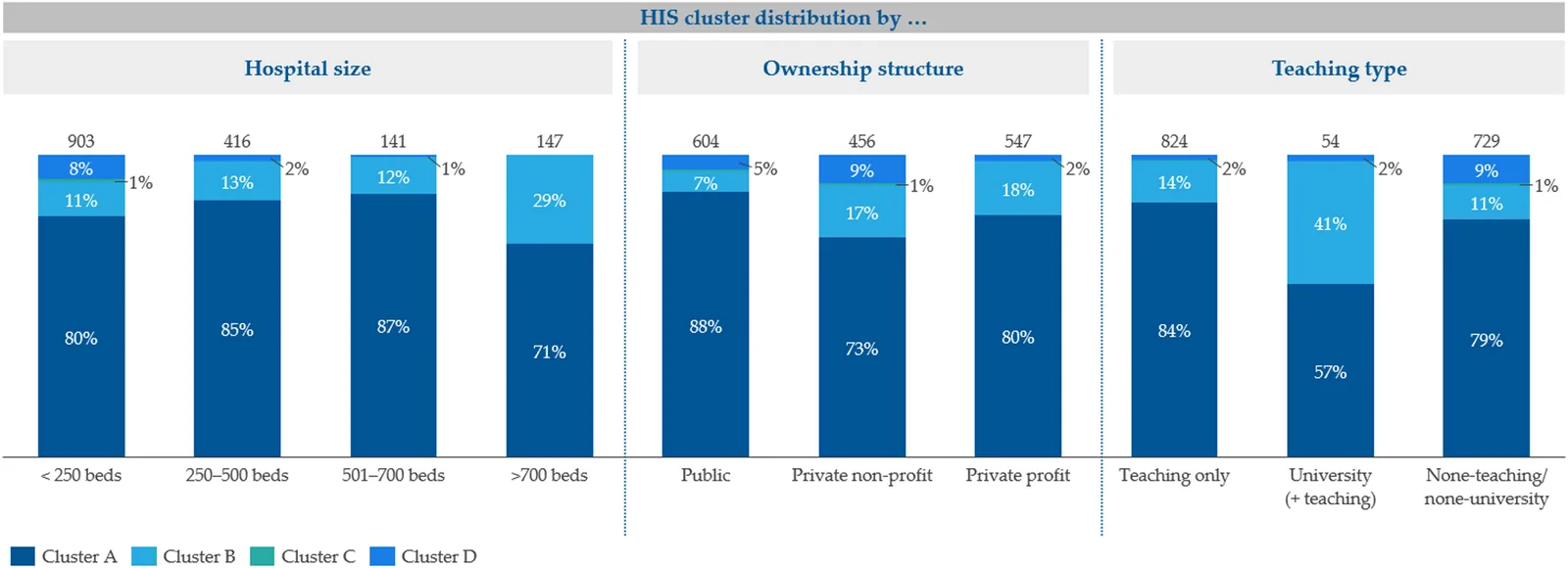
HIS cluster landscape stratified by hospital size, teaching type, and ownership structure. Note: Each bar represents one stratum. Bar segments show the proportion of hospitals assigned to each HIS provider cluster within that stratum. All bars are indexed to 100%. The total number of hospitals per stratum is shown above each bar

The HIS provider landscape varies across hospital size, ownership, and teaching type (Fig. 2). Cluster A dominates across all size categories, with the highest share among mid-to-large hospitals (87%). Cluster C and Cluster D are concentrated in smaller hospitals (< 500 beds). Ownership stratification reveals higher cluster diversity in private non-profit hospitals, reflected by greater shares of Cluster B (17%) and Cluster D (9%). Furthermore, 88% of hospitals using Clusters C or D providers are either public or private non-profit, with only 12% operating for profit. Regarding teaching status, university hospitals stand out for their heavy reliance on providers with low *External Provider Variation* (Cluster B: 41%), in sharp contrast to teaching hospitals, which predominantly choose Cluster A (84%). Notably, university hospitals rarely select providers with low integration ratio (Cluster C) or moderate *Module Utilization* (Cluster D). Regional variation was also evident (Supplementary Material 7). Bremen, Mecklenburg-Western Pomerania, and Thuringia reported no hospitals using Clusters C or D, while Hamburg, Schleswig-Holstein, and Bavaria showed the highest shares of hospitals using providers with low integration ratio or limited *Module Utilization*.

### Regression Outcomes

We first assessed the association between the specific HIS provider and the total DR-score using OLS regression (Supplementary Material 8). Most HIS providers exhibited significantly lower maturity scores compared to the reference (Provider 1), motivating further exploration of the underlying HIS features.

Diagnostic checks identified skewed residuals for *Patient Participation* and *Discharge* scores, which were square-root transformed (Supplementary Materials 9 to 10). No problematic multicollinearity among predictors was observed using VIF (Supplementary Material 11). Heteroscedasticity tests indicated unequal variances, requiring the use of robust standard errors (Supplementary Material 12).

For the primary outcome (i.e., total DR-score), *Module Utilization* (β = 6.871, *p* < 0.05) and *Integration Ratio* (β = 14.348, *p* < 0.01) emerged as positive predictors (Table 4). Meanwhile, *External Provider Variation* and *Maximum Hospital Size Coverage* showed no significant association.

**Table 4:** Ordinary least squares regression results with HIS features as predictors

Dependent variables	Total	DR dimensional scores							DR workflow scores			
	DR- score	Structures & Systems	Resilience	Organizati-onal Control	Clinical Processes	Information Exchange	Telehealth	SQRT(Patient Participation)	Admin-istrative	Admission	Inpatient Treatment	SQRT (Discharge)
Model	**(1)**	**(D1)**	**(D2)**	**(D3)**	**(D4)**	**(D5)**	**(D6)**	**(D7)**	**(W1)**	**(W2)**	**(W3)**	**(W4)**
	Coefficient (robust SE)	Coefficient (robust SE)	Coefficient (robust SE)	Coefficient (robust SE)	Coefficient (robust SE)	Coefficient (robust SE)	Coefficient (robust SE)	Coefficient (robust SE)	Coefficient (robust SE)	Coefficient (robust SE)	Coefficient (robust SE)	Coefficient (robust SE)
**Predictors**												
HIS features												
Module Utilization	6.871** (2.948)	12.339***^†^ (4.559)	8.429** (4.267)	8.370**^†^ (3.629)	7.666* (4.123)	2.900 (2.862)	−5.287 (3.819)	−0.036 (0.428)	7.937**^†^ (3.559)	−1.769 (3.412)	7.466**^†^ (3.557)	−0.339 (0.437)
Integration Ratio	14.348*** (3.786)	29.415***^†††^ (5.873)	11.246**^††^ (5.008)	12.158**^††^ (5.227)	17.038***^†††^ (5.439)	6.786* (3.703)	4.299 (5.302)	0.067 (0.604)	11.917***^††^ (4.569)	5.767 (4.439)	15.854***^†††^ (4.642)	0.325 (0.685)
Ext. Provider Variation	0.001 (0.012)	−0.008 (0.018)	−0.016 (0.019)	−0.004 (0.014)	0.019 (0.016)	0.006 (0.012)	0.018 (0.015)	−0.001 (0.002)	−0.009 (0.015)	0.014 (0.013)	0.013 (0.014)	0.002 (0.002)
Max. Hospital Size	−0.001 (0.001)	−0.002 (0.001)	−0.002 (0.001)	−0.001 (0.001)	−0.001 (0.001)	0.001 (0.001)	0.002* (0.001)	0.000 (0.000)	−0.001 (0.001)	0.001 (0.001)	−0.001 (0.001)	0.000 (0.000)
**Control variables**												
Teaching	3.217*** (0.522)	6.753*** (0.876)	1.436* (0.823)	2.041*** (0.673)	4.583*** (0.745)	1.674*** (0.55)	4.478*** (0.749)	0.125 (0.083)	2.032*** (0.668)	4.403*** (0.591)	3.608*** (0.655)	0.178** (0.082)
University	−0.594 (1.488)	−2.958 (2.059)	1.358 (2.337)	2.110 (1.840)	−3.304 (2.176)	0.545 (1.565)	2.810 (2.267)	0.399 (0.289)	1.337 (1.893)	0.181 (1.64)	−1.342 (1.973)	−0.235 (0.207)
Psychiatric	2.148*** (0.777)	4.559*** (1.274)	5.024*** (1.068)	2.438** (1.075)	1.393 (1.138)	0.477 (0.771)	0.830 (1.155)	0.042 (0.108)	3.805*** (0.912)	−1.067 (0.925)	1.491 (1.008)	−0.263** (0.112)
* Ownership base: Public*												
Ownership (private non-profit)	−1.452 (1.136)	−5.351*** (1.761)	1.483 (1.735)	−0.549 (1.394)	−2.974* (1.564)	−0.312 (1.123)	−1.909 (1.324)	0.129 (0.155)	0.142 (1.441)	−1.837 (1.131)	−1.895 (1.381)	0.176 (0.157)
Ownership (private for-profit)	1.780** (0.906)	3.820*** (1.427)	−0.838 (1.433)	0.297 (1.156)	3.408*** (1.245)	1.209 (0.986)	5.008*** (1.166)	−0.115 (0.128)	−0.568 (1.164)	4.032*** (0.957)	2.892*** (1.099)	0.100 (0.135)
Number of beds	0.008*** (0.001)	0.017*** (0.002)	0.004*** (0.002)	0.006*** (0.001)	0.010*** (0.001)	0.004*** (0.001)	0.005*** (0.001)	0.001*** (0.000)	0.005*** (0.001)	0.007*** (0.001)	0.009*** (0.001)	0.001*** (0.000)
IT FTE per bed (log)	5.834*** (1.275)	8.126*** (2.014)	8.623*** (1.911)	5.83*** (1.541)	5.547*** (1.763)	2.531** (1.279)	3.435** (1.661)	0.333* (0.192)	7.227*** (1.591)	1.772 (1.404)	5.273*** (1.566)	0.144 (0.195)
HIS provider module share	4.324*** (1.335)	6.213*** (2.062)	3.416* (1.916)	3.909** (1.664)	7.197*** (1.86)	0.254 (1.239)	0.393 (1.537)	−0.130 (0.174)	3.231** (1.618)	0.825 (1.327)	6.013*** (1.632)	−0.030 (0.187)
Federal state	Yes	Yes	Yes	Yes	Yes	Yes	Yes	Yes	Yes	Yes	Yes	Yes
Hospital chain	Yes	Yes	Yes	Yes	Yes	Yes	Yes	Yes	Yes	Yes	Yes	Yes

*N* = 1,607. Robust standard errors are shown in parentheses. Numbers smaller than 0.001 are displayed as 0.000. Significance is reported with raw p-values for the primary outcome (DR-score total) and with both raw p-values and Benjamini-Hochberg adjusted q-values within each predictor family for secondary outcomes (DR-score dimensions D1–D7 and workflow scores W1–W4). Significance for control variables is reported with raw p-values only. The following significance levels apply: *** *p* < 0.01, ** *p* < 0.05, * *p* < 0.10; ††† q < 0.01, †† q < 0.05, † q < 0.10

For the secondary outcomes (i.e., dimensional and workflow scores), *Module Utilization* and *Integration Ratio* show the most consistent positive associations, robust to FDR correction. As such, *Module Utilization* is positively associated with digital maturity scores for *Structures and Systems* (β = 12.339, *p* < 0.01), *Clinical Processes* (β = 7.666, *p* < 0.1), and *Inpatient Treatment* workflows (β = 7.466, *p* < 0.05). Similarly, a higher *Integration Ratio* shows robust positive associations with *Structures and Systems* (β = 29.415, *p* < 0.01), *Clinical Processes* (β = 17.038, *p* < 0.01), and *Inpatient Treatment* workflows (β = 15.854, *p* < 0.01).

As a supplementary analysis to our HIS clustering, we re-ran regressions using HIS clusters as a categorical predictor (Supplementary Material 13). Hospitals with Cluster C and D providers were consistently associated with lower maturity, while differences between Cluster A and B were small. Given the small size of Cluster C (*N* = 7), cluster-specific regressions should be interpreted cautiously.

In line with Geissler et al. [24], teaching status and hospital size (bed count) were positively associated with digital maturity, while private ownership (non-profit and for-profit) showed selective positive effects. IT FTE per hospital bed was also positively associated across DR-score and dimensions. Given the mean of 0.02 IT FTE per bed (≈ 2 per 100 beds) (Table 2), doubling the IT FTE per bed is associated with a 4.0-point higher DR-score (ln(2) = 0.693; 5.834 × 0.693 ≈ 4.0), holding all else constant.

Lastly, the *HIS provider module share* was also positively associated with digital maturity (β = 4.324, *p* < 0.01), indicating higher digital maturity in hospitals whose module portfolio is more strongly concentrated on their HIS provider.

### Sensitivity Analyses

We assessed whether including *Cloud Offering* as an additional feature affects clustering and regression results (Supplementary Material 14–21). Upon inclusion, some providers shifted between clusters and a new Cluster E emerged; however, results were largely consistent with the base model. I*ntegration Ratio* remained a strong predictor of digital maturity, whereas *Cloud Offering* itself was not significantly associated with the overall DR-score. Cluster-level patterns were also unchanged, with Clusters A and B performing similarly and Clusters C, D, and E showing lower maturity. Lastly, results remained consistent when excluding the *Structures and Systems* dimension, with key predictors retaining direction and significance (Supplementary Material 22).

## Discussion

This study assessed whether the choice of HIS provider is associated with hospitals’ digital maturity. We found that HIS provider selection is associated with digital maturity. Specifically, providers with broad *Module Utilization* and a strong integration ratio were linked to higher maturity scores.

### Importance of Interoperability and Module Utilization

Interoperability is commonly defined as a system’s ability to exchange health data across systems, providers, and regional/national boundaries. In this study, we used the *Integration Ratio* as a proxy, which emerged as one of the strongest predictors of digital maturity, particularly in *Structures and Systems*, *Clinical Processes*, and *Organizational Control*. In practice, integrated systems break data silos by ensuring captured data become rapidly available across modules, translating into streamlined clinical workflows (e.g., avoiding repeated data entry) [38], reduced diagnostic redundancies, and improved governance and quality reporting [39].

However, gaps remain between ambition and practice. Teixeira et al. [40] found that although 90% of general practitioners across 20 countries reported routine EHR use, only 47% considered their systems interoperable. Similarly, Gheorghiu and Hagens [41] documented that even where interoperable systems were technically available, integration into workflows remained limited. Thus, while most providers claim interoperability, the *Integration Ratio* captures the integration HIS providers actually achieve in practice.

In a 2023 U.S. survey, 96% of hospitals reported at least one barrier to interoperable exchange, and 62% cited major barriers. The most common were vendor-related incompatibility (84% of hospitals) and difficulties in patient matching (57%). Costly data-sharing arrangements and mismatched patient identifiers showed the strongest negative associations with routine use of external information [38].

While we applied multiple HIS features to cluster providers, prior research often classifies HIS as monolithic (i.e., systems providing most modules within one platform) or best-of-breed (i.e., multi-vendors combining systems via interfaces) [10, 30, 42, 43]. Monolithic solutions offer simpler maintenance, seamless integration, and smoother data flows, whereas best-of-breed environments provide flexibility but risk fragmented data and higher integration costs. The downsides of monolithic approaches are well recognized and include, for example, workflow rigidity or vendor lock-in [10].

Our findings show that hospitals deploying HIS with higher *Module Utilization* achieved significantly higher overall maturity scores, with the strongest gains in inpatient treatment workflows. Additionally, hospitals whose module portfolio was more strongly concentrated on their HIS provider showed higher digital maturity, independent of that provider’s characteristics. In practice, we hypothesize that a broad module offering by the HIS provider creates the architectural opportunity for a monolithic setup, while a high share of modules sourced from the HIS provider indicates that this opportunity has been realized. Together, this may support hospitals in sharing consistent data structures and user interfaces, translating into broader system functionality, fewer fragmented workflows, easier cross-departmental work, and more mature digital infrastructure. EHR studies show that hospitals with comprehensive module integration (e.g., laboratory, pharmacy, and imaging functions) are more likely to reach advanced Electronic Medical Record Adoption Model (EMRAM) stages, an eight-stage framework assessing the digital maturity of acute somatic care institutions [44]. Similarly, Hamadi et al. [30] found that monolithic systems users were more likely to implement patient safety protocols, follow clinical guidelines, and use organizational dashboards. In contrast, fragmented best-of-breed environments often hinder these outcomes [45]. Looking forward, Vest et al. [43] predict a continued shift toward monolithic strategies.

Both *External Provider Variation* and *Maximum Hospital Size Coverage* showed mixed evidence. We hypothesize that *External Provider Variation* has both beneficial and adverse effects. It may contribute to maturity in domains where third-party solutions are common, such as *Clinical Process*, but also increases interface and coordination complexity, potentially offsetting maturity gains via domains such as IT resilience or administrative effort. *Maximum Hospital Size Coverage* reflects an HIS’s ability to operate in large, complex hospital environments. However, more than 50% of hospitals in our sample have 250 beds or fewer. Capabilities designed for highly complex settings may therefore be less relevant for most hospitals, which require solutions tailored to smaller organizational environments.

### Challenges with System Transition

Hospitals may face barriers that hinder transitions to HIS providers with broader module coverage and stronger integration ratio. Huang et al. [46] identify common reasons hospitals retain existing systems, including high costs, staffing constraints, technical requirements (e.g., hardware, data migration), patient safety risks, clinician resistance, negative patient experience, inadequate training, cybersecurity, and leadership challenges. Similar dynamics apply to HIS transitions. Wetter [47] highlights that interconnected HIS architectures make even partial replacements risky, with errors (e.g., patient ID mismatches) that can cascade into broader failures. Consequently, decisions about whether to maintain or replace systems are complex. Chryssanthou et al. [48] proposed an eight-factor framework to guide replacement strategies, including factors such as error rates, productivity, popularity, and user feedback.

### Practical Implications

Previous literature defined digital maturity as the extent to which digitalization is employed to deliver high-quality care [40]. While our study focuses on digital maturity itself, these levels have real-world implications for patients. Snowdon et al. [44] analyzed 1,026 U.S. hospitals and found that institutions at advanced EMRAM levels had 3.25 times higher odds of achieving a top Leapfrog Safety Grade, including fewer adverse events, lower infection rates, and improved surgical safety. Benefits materialized via automated data flows between departments, better safety tracking, and a strong safety culture. Chaudhry et al. [49] reported similar effects in a systematic review, noting that health information technology increases adherence to guidelines, enhances survival and monitoring, and decreases medication errors. Importantly, the greatest effects were seen in complex, high-risk cases (e.g., pneumonia, coronary atherosclerosis), which require extensive cross-specialty care and complex information flow [50]. However, technology alone is insufficient. Von Wedel et al. [11] argue that physicians and nurses must perceive systems as valuable. Their study found that a one-point increase in user-perceived value was correlated with a 3.15% reduction in stroke mortality.

Hospitals should treat HIS selection as a strategic decision with implications for digital maturity. Procurement processes should evaluate provider characteristics such as module utilization, interoperability, and openness to external systems, thereby rewarding vendors that enable rather than constrain digital transformation. We recognize that a complete system replacement is often neither operationally nor financially feasible. This is particularly true for hospitals in Clusters C and D, which tend to have fewer beds and may lack the resources required for large transformations. For these hospitals, improving digital maturity without a full system overhaul remains essential. Importantly, our examined HIS features explain only part of the variation in digital maturity. Hence, several measures may be implemented independently of the underlying HIS, including promoting patient portal enrollment, regularly testing outage response plans, providing cybersecurity awareness training, harmonizing clinical and IT workflows, and developing a digital strategy that explicitly links IT investments to long-term organizational objectives [51, 52]. Hospitals may further enhance interoperability through integration engines or FHIR-based application programming interfaces (APIs) rather than replacing existing platforms [52]. Where technically feasible, they may also activate unused software modules already included in their HIS provider’s product suite. Finally, implementing these measures requires sufficient IT capacity. Our findings show that IT FTEs per bed are strongly associated with DR-scores, suggesting that IT staffing is an important lever for advancing digital maturity.

Policymakers should recognize that DR-scores reflect both hospital efforts and constraints in the HIS ecosystem. Monitoring provider-level differences and incentivizing transitions away from systems with low module utilization and integration ratio could accelerate digitization. U.S. programs such as Medicare’s Promoting Interoperability and TEFCA illustrate how nationwide data exchange can be fostered. Yet, some argue that incentives alone may be insufficient. Stronger enforcement against information blocking (i.e., providers knowingly restricting data exchange) could establish interoperability as a baseline expectation rather than a competitive advantage [53]. Public reporting, as exemplified by the ONC Certified Health IT Product List [54], could further strengthen procurement negotiations and incentivize vendor improvements.

Germany’s HIS market is crowded, with 24 providers active. Providers that expand module utilization and deliver robust interoperability can differentiate and position themselves as key enablers of digital transformation. As Wang et al. [55] noted, shifting from proprietary to open interfaces may alter market-share strategies, but can accelerate innovation and diversify product risk.

### Limitations

This study has limitations. First, we identified HIS providers but lacked information on the exact systems deployed, which may vary in functionality. Additionally, our four HIS features reflect providers rather than individual deployments. While this design aligns with our research question on HIS provider choice, it does not capture within-provider heterogeneity (i.e., hospitals using the same HIS provider may differ in module configuration or integration status). For instance, the Integration Ratio reflects observed integration outcomes, which may be jointly shaped by an HIS provider’s technical openness and by hospital-specific factors (e.g., individual investment in middleware or contractual agreements) that our data cannot fully disentangle. Our model partially accounts for hospital-level integration capacity through the inclusion of IT FTE per bed as a control variable, though numerous additional influencing factors may lie outside the scope of our dataset. Nevertheless, to our knowledge, the DR is the most comprehensive dataset on the HIS provider landscape in Germany, covering nearly all hospitals. Second, clustering was based on a limited number of providers, yielding instability in Cluster B. However, since Clusters A and B share similar characteristics, merging them would not materially alter the interpretation of our findings. Third, our primary cluster solution is unbalanced at the hospital level, with Cluster C comprising only seven hospitals. Although bootstrapping confirmed moderate stability, estimates for Cluster C should be interpreted as directional rather than precise. Fourth, our analysis relies on self-reported survey data without independent verification. Yet, as survey completion was required for Hospital Future Act funding, hospitals had a strong incentive to provide accurate and reliable information.

Future research should leverage longitudinal DR data from 2024 and further DR assessments to test whether hospitals using certain HIS achieve greater maturity improvements over time, and in which DR-score domains. Further studies should also examine whether higher digital maturity translates into improved quality and patient outcomes. Moreover, hospital-level analyses offer opportunities for future research to capture within-provider heterogeneity. Finally, as HIS usability and interoperability challenges extend beyond Germany, cross-country comparisons may clarify whether the association between HIS choice and digital maturity is context-specific or systemic.

## Conclusion

Our study showed that HIS provider choice is significantly associated with hospitals’ digital maturity in Germany. Providers with broad *Module Utilization* and a strong *Integration Ratio* were linked to higher maturity scores.

## Supplementary Information

Below is the link to the electronic supplementary material.

Supplementary Material (DOCX 893 KB)

## Data Availability

Data used in this study are available from the FDZ DigitalRadar Krankenhaus (DiRa-K) dataset provided by the RWI – Leibniz-Institut für Wirtschaftsforschung (Haering & Hollenbach, 2025). Access can be requested at https://www.rwi-essen.de/fileadmin/user_upload/RWI/FDZ/FDZ_Datensatzbeschreibung_DigitalRadar.pdf. Data related to specific Hospital Information System providers are not publicly available due to restrictions on ownership by the German Federal Ministry of Health. However, data are available upon reasonable request.

## Code Availability

The code is available from the corresponding author upon request.
