# Supplementary material for: Does System Choice Matter? Influence of Hospital Information Systems on Digital Maturity
Source: J Med Syst. 2026 Aug 8;50(1):124. doi: 10.1007/s10916-026-02450-w (PMC13452863; doi:10.1007/s10916-026-02450-w)
Supplement: Supplementary file 1 [file 10916_2026_2450_MOESM1_ESM.docx]

Supplementary Material for:

Does System Choice Matter? Influence of Hospital Information Systems on Digital Maturity

Jonas Backes^1, C^; Alexander Geissler^1^; Jonas Subelack^1^; David Ehlig^1^

^1^ Chair of Health Economics, Policy and Management; School of Medicine; University of St. Gallen;
St. Gallen; Switzerland

^C^ Corresponding author, jonas.backes@unisg.ch

Supplementary Material 1. Overview of hospital-specific data variables extracted from the DigitalRadar (DR) survey

| **Data categories** | | **Data variables** | **Variable reflects the...** | **Scale** | **Data type** |
| --- | --- | --- | --- | --- | --- |
| **Digital maturity** | **Total** | DR-score | … hospitals’ overall digital maturity via an aggregated score based on DR-score dimensions | Interval | Continuous (0–100) |
|  | **DR-score dimensions** | Structures and Systems | … capacity, staffing, and financial status, and the availability of software applications | Interval | Continuous (0–100) |
|  |  | Resilience Mgmt. and Performance | … hospital-level IT security, agility, and resilience to internal and external challenges |  |  |
|  |  | Org. Control and Data Management | … ability of existing structures and processes to support strategic use of digital tools |  |  |
|  |  | Clinical  Processes | … effectiveness of digital tools in supporting clinical processes |  |  |
|  |  | Information Exchange | … ability of the hospital to securely communicate clinical data |  |  |
|  |  | Telehealth | … existence of a virtual care model in diagnoses, treatment, and rehabilitation |  |  |
|  |  | Patient Participation | … empowerment of patients to access and manage data, and engage via digital tools |  |  |
|  | **Workflow** | Administrative | … digital maturity of administrative workflows | Interval | Continuous (0–100) |
|  |  | Admission | … digital maturity of admission workflows |  |  |
|  |  | Inpatient  Treatment | … digital maturity of inpatient treatment |  |  |
|  |  | Discharge | … digital maturity of discharge workflows |  |  |
| **Hospital  description** | | Hospital  type | … classification of the hospital based on its role in medical education and care focus | Nominal | Discrete (teaching; university; psychiatric) |
|  |  | Ownership | … hospital’s legal ownership structure | Nominal | Discrete (public; private non-profit; private for-profit) |
|  |  | Hospital  size | … number of beds per hospital | Ratio | Continuous  (≥0) |
|  |  | IT department  size | … IT full time equivalents (FTE) per hospital bed for each hospital | Ratio | Continuous  (≥0) |
|  |  | HIS provider module share | … share of all modules present at a hospital  that are supplied by its primary HIS provider | Ratio | Continuous (0–1) |
|  |  | Hospital chain | … specific multi-hospital group operating the hospital, or operation as an individual hospital | Nominal | Discrete  (individual hospital; 193 hospital chains) |
| **Geographic description** | | Federal  state | … federal state in which the hospital is located | Nominal | Discrete  (16 federal states) |

Note: All data were collected in 2021. A more detailed description of the dimensions can be found in the results of the digital maturity assessment by Geissler et al. [24].

Supplementary Material 2. Overview of potential hospital IT sub-systems covered in the nationwide DigitalRadar (DR) survey

| **#** | **Module names** | **Running licenses^1^** |
| --- | --- | --- |
| 1 | Anatomical Pathology | 196 |
| 2 | Cardiology Information System | 656 |
| 3 | Cardiology Picture Archiving and Communication System (PACS) | 615 |
| 4 | Clinical Documentation | 1341 |
| 5 | Communication Server | 1280 |
| 6 | Computerized Practitioner Order Entry (CPOE) | 1386 |
| 7 | Electronic Medication Administration Record (EMAR) | 628 |
| 8 | Emergency Department Information System (EDIS) | 754 |
| 9 | ePrescribing | 650 |
| 10 | Laboratory Information System (LIS) | 991 |
| 11 | Patient Data Management System (PDMS) | 500 |
| 12 | Patient Portal | 72 |
| 13 | Pharmacy Management System | 556 |
| 14 | Physician Documentation | 1519 |
| 15 | Radiology Information System (RIS) | 949 |
| 16 | Radiology Picture Archiving and Communication System (PACS) | 1105 |
| 17 | Vendor Neutral Archive (VNA) | 857 |

Note: The IT systems listed represent the modules covered by the nationwide DigitalRadar survey (N = 1,607) in 2021. 1. Running licenses are those that the hospital currently indicates as live and operational.

Supplementary Material 3. Overview of HIS variable results per provider

|  |  | HIS features |  |  |  |  |
| --- | --- | --- | --- | --- | --- | --- |
|  |  | Base model |  |  |  | Sensitivity |
|  |  | 1. | 2. | 3. | 4. | 5. |
| HIS  ID | Running license(s) | Module Utilization  (absolute) | Integration Ratio | External Provider Variation | Maximum  Hospital Size Coverage^1^ | Cloud  Offering |
| 1 | 601 | 1.00 (17) | 0.70 | 98 | >700 | Yes |
| 2 | 312 | 0.94 (16) | 0.70 | 87 | >700 | No |
| 3 | 23 | 0.47 (8) | 0.57 | 17 | 250–500 | Yes |
| 4 | 11 | 0.29 (5) | 0.60 | 2 | <250 | No |
| 5 | 13 | 0.18 (3) | 0.71 | 10 | 501–700 | No |
| 6 | 120 | 0.65 (11) | 0.83 | 71 | >700 | No |
| 7 | 11 | 0.88 (15) | 0.74 | 10 | 501–700 | No |
| 8 | 18 | 0.47 (8) | 0.61 | 9 | 250–500 | No |
| 9 | 1 | 0.06 (1) | 0.50 | 1 | <250 | No |
| 10 | 3 | 0.18 (3) | 0.00 | 0 | <250 | No |
| 11 | 6 | 0.24 (4) | 0.71 | 8 | 250–500 | No |
| 12 | 68 | 0.82 (14) | 0.76 | 0 | >700 | Yes |
| 13 | 149 | 0.94 (16) | 0.60 | 68 | >700 | Yes |
| 14 | 1 | 0.24 (4) | 0.33 | 1 | <250 | No |
| 15 | 41 | 0.53 (9) | 0.84 | 42 | >700 | No |
| 16 | 2 | 0.00 (0) | 0.00 | 0 | <250 | No |
| 17 | 78 | 0.71 (12) | 0.75 | 53 | >700 | No |
| 18 | 2 | 0.00 (0) | 0.50 | 2 | <250 | Yes |
| 19 | 138 | 0.88 (15) | 0.85 | 0 | >700 | No |
| 20 | 1 | 0.06 (1) | 0.75 | 3 | <250 | Yes |
| 21 | 2 | 0.24 (4) | 1.00 | 4 | <250 | No |
| 22 | 2 | 0.06 (1) | 0.75 | 1 | 250–500 | No |
| 23 | 3 | 0.41 (7) | 0.76 | 8 | 250–500 | No |
| 24 | 1 | 0.00 (0) | 0.00 | 0 | <250 | No |
|  |  |  |  |  |  |  |
| Mean^2^ | 66.96 | 0.43 (7.31) | 0.60 | 20.63 | 64.96 | 0.25 |

Note: 1. To avoid re-identification of HIS providers, Maximum Hospital Size Coverage is displayed as ranges only. All underlying analyses (i.e., k-means clustering, cluster centroids calculation, and regression analyses) use the absolute bed counts. 2. Mean for dummy variables calculated as proportion of 1 over total number of HIS providers (= 24). HIS variable values are calculated based on DigitalRadar hospital-level data (N = 1,607) aggregated at the provider level. Features 1 to 4 are derived directly from survey responses. Variable 5 is extracted based on publicly available information. Values are used for k-means clustering and are normalized before clustering.

Supplementary Material 4. Total within-cluster sum of squares across different numbers of clusters (k) for base modeling

Note: The plot shows the total within-cluster sum of squares for different numbers of HIS provider clusters. K = 1 represents only one group of 24 HIS providers. The inflection point indicates the optimal number of clusters based on the trade-off between complexity and variance.

Supplementary Material 5. Average silhouette score across different numbers of clusters (k) for base modeling

Note: The graph shows the quality of HIS provider clustering by how well each provider fits within its own cluster relative to other clusters. Scores closer to 1 indicate stronger separation, while scores close to zero indicate strong overlapping clusters. Each number of clusters was tested using 50 random initializations to improve stability.

Supplementary Material 6. Within-cluster sum of squares (WSS), WSS reduction (ΔWSS), and average silhouette score across k = 2 to 10 for the base clustering model

| **K** | **WSS** | **ΔWSS (relative to k − 1)** | **Average silhouette** |
| --- | --- | --- | --- |
| 1 | 92.0 | – | – |
| 2 | 42.0 | −50.0 | 0.476 |
| 3 | 27.2 | −14.8 | 0.454 |
| 4 | 17.4 | −9.8 | 0.476 |
| 5 | 13.3 | −4.1 | 0.398 |
| 6 | 10.2 | −3.1 | 0.374 |
| 7 | 7.5 | −2.7 | 0.399 |
| 8 | 6.0 | −1.5 | 0.388 |
| 9 | 4.6 | −1.4 | 0.387 |
| 10 | 3.7 | −0.9 | 0.387 |

Note: WSS decreases with k by construction. The elbow is identified at k = 4, as the reduction in WSS more than halves (from −9.8 to −4.1) and plateaus for k ≥ 5. Silhouette scores closer to 1 indicate stronger separation. K = 1 has no silhouette score by definition.

Supplementary Material 7. HIS cluster landscape stratified by federal state

Note: Each bar represents one federal state. The height of each bar segment indicates the proportion of hospitals assigned to the corresponding HIS provider cluster. Percentages within bars represent the proportion of each HIS provider cluster in the respective state. The total number of hospitals per state is shown at the top of each bar. All bars are indexed to 100 percent.

Supplementary Material 8. Ordinary least squares regression results for the effect of HIS provider on total DR-score

|  | **Dependent variable: total DR-score** | |
| --- | --- | --- |
|  | **(1) Unadjusted model** | **(2) Adjusted model** |
|  | Coefficient  (robust SE) | Coefficient  (robust SE) |
| **Predictors** |  |  |
| HIS provider ID (Base: 1) |  |  |
| 2 | -2.883***  (0.757) | -0.233  (0.962) |
| 3 | -14.431***  (2.513) | -7.705**  (3.090) |
| 4 | -13.971***  (1.853) | -7.59***  (1.636) |
| 5 | -7.490***  (1.829) | -0.982  (3.756) |
| 6 | 2.116**  (0.974) | 1.819  (1.175) |
| 7 | -2.694  (3.639) | 7.477  (5.180) |
| 8 | -6.910***  (2.027) | -4.204  (3.026) |
| 9 | -23.439***  (0.415) | -16.303***  (2.121) |
| 10 | -16.858***  (3.906) | -11.961***  (4.472) |
| 11 | -14.441***  (5.197) | 0.478  (5.891) |
| 12 | -4.614***  (1.263) | 0.028  (1.542) |
| 13 | 1.062  (0.852) | 0.845  (1.363) |
| 14 | -4.699***  (0.415) | -1.884  (1.617) |
| 15 | 0.399  (1.913) | 0.140  (1.741) |
| 16 | -23.923***  (2.020) | -15.815***  (1.928) |
| 17 | -3.997***  (1.158) | -1.252  (1.199) |
| 18 | -15.256***  (3.925) | -4.684  (4.751) |
| 19 | 4.889***  (1.002) | 1.010  (1.18) |
| 20 | -10.069***  (0.415) | -6.744**  (2.624) |
| 21 | -8.710**  (3.676) | -17.124***  (4.099) |
| 22 | -18.113***  (0.682) | -4.613*  (2.504) |
| 23 | 2.693  (6.578) | -3.797  (4.552) |
| 24 | -18.633***  (0.415) | -3.895*  (2.080) |
| Control variables |  |  |
| Teaching |  | 3.231***  (0.53) |
| University |  | -0.439  (1.497) |
| Psychiatric |  | 2.091***  (0.776) |
| Ownership base: Public |  |  |
| Ownership (private non-profit) |  | -1.379  (1.182) |
| Ownership (private for-profit) |  | 1.629*  (0.915) |
| Number of beds |  | 0.008***  (0.001) |
| IT FTE per bed (log) |  | 6.248***  (1.32) |
| HIS provider module share |  | 4.452***  (1.379) |
| Federal state |  | Yes |
| Hospital chain |  | Yes |

Note: N = 1,607. Robust standard errors are shown in parentheses. The dependent variable is the total DR-score. Model (1) is unadjusted, including only HIS provider ID as a predictor (Base: Provider 1). Model (2) is adjusted, additionally including hospital-level control variables (teaching status, university hospital, psychiatric hospital, ownership, number of beds, log IT FTE per bed, federal state, and hospital chain fixed effects). Numbers are rounded to three decimal places. The following significance levels apply: *** p < 0.01, ** p < 0.05, * p < 0.10.

Supplementary Material 9. Quantile-Quantile (Q-Q) plots of residuals for regression models with HIS features as predictors

Note: Quantile-Quantile (Q-Q) plots for outcome variables compare the observed distribution of residuals (y-axis) with the expected distribution of residuals if the data were perfectly normally distributed (x-axis). The red diagonal line shows an ideal normal distribution. Points deviating from the line indicate a violation of the normality assumption.

Supplementary Material 10. Histograms of residuals for regression models with HIS features as predictors

Note: Histograms show the distribution of regression model residuals with the HIS features as predictors. The x-axis represents residual values. The y-axis indicates the frequency of residual values.

Supplementary Material 11. Variance Inflation Factors (VIF) for predictors in regression models with HIS features and HIS clusters

| **Predictors** | **VIF (HIS features model)** | **VIF (HIS cluster model)** |
| --- | --- | --- |
| Module Utilization | 2.28 |  |
| Integration Ratio | 1.64 |  |
| External Provider Variation | 1.80 |  |
| Maximum Hospital Size Coverage | 2.27 |  |
| HIS Cluster |  | 1.22 |
| Teaching | 1.34 | 1.34 |
| University | 1.30 | 1.30 |
| Psychiatric | 1.30 | 1.29 |
| Ownership | 1.74 | 1.74 |
| Number of beds | 1.50 | 1.49 |
| IT FTE per bed (log) | 1.42 | 1.42 |
| HIS provider module share | 1.37 | 1.30 |
| Federal state | 1.21 | 1.20 |
| Hospital chain | 1.03 | 1.03 |

Note: The commonly accepted VIF range of 1 to 5 is used as a threshold. Values below 5 indicate low multicollinearity. All predictors are within this range.

Supplementary Material 12. Breusch-Pagan (BP) test for heteroscedasticity in regression models with HIS features and HIS clusters as predictors

|  | HIS feature predictor | | | HIS cluster predictor | | |
| --- | --- | --- | --- | --- | --- | --- |
| Outcome variable | BP statistic | df | p-value | BP statistic | df | p-value |
| DR-score | 323.75 | 2019 | <0.001 | 306.53 | 2018 | <0.001 |
| **DR-score dimension** |  |  |  |  |  |  |
| Structures & Systems | 315.04 | 2019 | <0.001 | 301.7 | 2018 | <0.001 |
| Resilience Mgmt. & Performance | 353.75 | 2019 | <0.001 | 351.29 | 2018 | <0.001 |
| Organizational Control & Data Mgmt. | 322.04 | 2019 | <0.001 | 316.07 | 2018 | 0.001 |
| Clinical Processes | 330.86 | 2019 | <0.001 | 317.52 | 2018 | <0.001 |
| Information Exchange | 262.86 | 2019 | <0.001 | 259.54 | 2018 | <0.001 |
| Telehealth | 230.57 | 2019 | 0.283 | 213.61 | 2018 | 0.571 |
| Patient Participation | 370.91 | 2019 | <0.001 | 385.72 | 2018 | <0.001 |
| SQRT(Patient Participation) | 310.76 | 2019 | <0.001 | 318.6 | 2018 | <0.001 |
| **DR workflow scores** |  |  |  |  |  |  |
| Administrative | 372.03 | 2019 | <0.001 | 366.82 | 2018 | <0.001 |
| Admission | 212.7 | 2019 | 0.607 | 211.88 | 2018 | 0.604 |
| Inpatient Treatment | 325.06 | 2019 | <0.001 | 314.53 | 2018 | <0.001 |
| Discharge | 372.03 | 2019 | <0.001 | 262.11 | 2018 | <0.001 |
| SQRT(Discharge) | 275.72 | 2019 | <0.001 | 274.84 | 2018 | <0.001 |

Note: BP = Breusch-Pagan; df = Degrees of freedom; Mgmt. = Management; SQRT = Square root. A significant p-value (<0.05) indicates heteroscedasticity.

**Supplementary Material 13.** Ordinary least squares regression results with HIS clusters as predictors

| **Dependent variables** | **Total** | **DR dimensional scores** | | | | | | | **DR workflow scores** | | | |
| --- | --- | --- | --- | --- | --- | --- | --- | --- | --- | --- | --- | --- |
|  | DR- score | Structures  & Systems | Resilience | Organizati-onal Control | Clinical  Processes | Information Exchange | Telehealth | SQRT(Patient Participation) | Admin-istrative | Admission | Inpatient Treatment | SQRT (Discharge) |
| **Model** | (1) | (D1) | (D2) | (D3) | (D4) | (D5) | (D6) | (D7) | (W1) | (W2) | (W3) | (W4) |
|  | Coefficient  (robust SE) | Coefficient  (robust SE) | Coefficient  (robust SE) | Coefficient  (robust SE) | Coefficient  (robust SE) | Coefficient  (robust SE) | Coefficient  (robust SE) | Coefficient  (robust SE) | Coefficient  (robust SE) | Coefficient  (robust SE) | Coefficient  (robust SE) | Coefficient  (robust SE) |
| **Predictors** |  |  |  |  |  |  |  |  |  |  |  |  |
| HIS clusters |  |  |  |  |  |  |  |  |  |  |  |  |
| *Base: Cluster A* |  |  |  |  |  |  |  |  |  |  |  |  |
| Cluster B | 0.624  (0.888) | 1.788 (1.324) | 1.199 (1.346) | 1.124  (1.079) | -0.369  (1.235) | 0.551 (0.889) | 0.472 (1.094) | 0.091  (0.144) | 1.154  (1.092) | 0.593 (0.935) | -0.325  (1.106) | 0.020  (0.128) |
| Cluster C | -10.360*** (2.986) | -17.800***^†††^ (3.961) | -8.372**^††^ (3.377) | -11.019**^†^ (4.602) | -13.913***^†††^ (3.835) | -7.556**^†^ (3.513) | -2.245 (3.767) | 0.634  (0.870) | -10.301***^††^ (3.601) | -2.103 (5.540) | -12.418***^†††^ (3.062) | 0.028 (0.781) |
| Cluster D | -5.735*** (1.517) | -9.149***^†††^ (2.275) | -4.836**^†^ (2.292) | -6.219***^†††^ (1.841) | -7.789***^†††^ (1.952) | -3.487**^†^ (1.490) | -0.275 (1.836) | -0.070  (0.194) | -5.447***^††^ (1.980) | -2.670* (1.513) | -6.514***^†††^ (1.766) | -0.239 (0.213) |
| Control variables |  |  |  |  |  |  |  |  |  |  |  |  |
| Teaching | 3.198*** (0.523) | 6.741*** (0.869) | 1.366* (0.819) | 2.003*** (0.672) | 4.571*** (0.746) | 1.693*** (0.555) | 4.593*** (0.749) | 0.122  (0.083) | 1.984*** (0.665) | 4.464*** (0.597) | 3.594*** (0.656) | 0.183** (0.082) |
| University | -0.585  (1.501) | -2.990 (2.086) | 1.378 (2.349) | 2.162  (1.853) | -3.311  (2.181) | 0.615 (1.574) | 2.804 (2.285) | 0.407  (0.289) | 1.379  (1.907) | 0.245 (1.645) | -1.375  (1.983) | -0.228 (0.208) |
| Psychiatric | 2.128*** (0.764) | 4.551*** (1.262) | 5.297*** (1.064) | 2.441** (1.062) | 1.257  (1.118) | 0.218 (0.772) | 0.424 (1.135) | 0.041  (0.107) | 3.928*** (0.902) | -1.473 (0.935) | 1.424  (0.992) | -0.298*** (0.112) |
| *Ownership base: Public* |  |  |  |  |  |  |  |  |  |  |  |  |
| Ownership  (private non-profit) | -1.239  (1.141) | -5.054*** (1.754) | 1.789 (1.745) | -0.302  (1.390) | -2.696* (1.563) | -0.299 (1.144) | -2.114 (1.320) | 0.131  (0.155) | 0.406  (1.448) | -1.923* (1.137) | -1.650  (1.384) | 0.172 (0.158) |
| Ownership  (private for-profit) | 1.711*  (0.901) | 3.664*** (1.420) | -0.924 (1.434) | 0.254  (1.154) | 3.348*** (1.240) | 1.230  (0.979) | 4.968*** (1.167) | -0.122  (0.127) | -0.632  (1.164) | 3.996*** (0.950) | 2.839*** (1.093) | 0.094 (0.135) |
| Number of beds | 0.008*** (0.001) | 0.017*** (0.002) | 0.004** (0.002) | 0.006*** (0.001) | 0.010*** (0.001) | 0.004*** (0.001) | 0.005*** (0.001) | 0.001*** (0.000) | 0.005*** (0.001) | 0.007*** (0.001) | 0.009*** (0.001) | 0.001***  (0.000) |
| IT FTE per bed (log) | 6.008*** (1.268) | 8.418*** (2.008) | 8.705*** (1.903) | 5.998*** (1.536) | 5.778*** (1.747) | 2.703** (1.280) | 3.636** (1.679) | 0.340*  (0.191) | 7.369*** (1.582) | 2.006 (1.409) | 5.442*** (1.557) | 0.167 (0.192) |
| HIS provider module share | 4.377***  (1.241) | 5.868*** (1.918) | 3.245* (1.770) | 4.293*** (1.555) | 7.553*** (1.720) | 0.733 (1.179) | 0.297 (1.493) | -0.158  (0.171) | 3.284** (1.496) | 1.046 (1.272) | 6.231*** (1.515) | -0.020 (0.178) |
| Federal state | Yes | Yes | Yes | Yes | Yes | Yes | Yes | Yes | Yes | Yes | Yes | Yes |
| Hospital chain | Yes | Yes | Yes | Yes | Yes | Yes | Yes | Yes | Yes | Yes | Yes | Yes |

Note: N = 1,607. Robust standard errors are shown in parentheses. Numbers smaller than 0.001 are displayed as 0.000. Significance is reported with raw p-values for the primary outcome (DR-score total) and with both raw p-values and Benjamini-Hochberg adjusted q-values within each predictor family for secondary outcomes (DR-score dimensions D1–D7 and workflow scores W1–W4). Significance for control variables is reported with raw p-values only. The following significance levels apply: *** p < 0.01, ** p < 0.05, * p < 0.10; ^†††^ q < 0.01, ^††^ q < 0.05, ^†^ q < 0.10.

Supplementary Material 14. Total within-cluster sum of squares across different numbers of clusters (k), including the Cloud Offering variable

Note: The plot shows the total within-cluster sum of squares for different numbers of HIS provider clusters. K = 1 represents only one group of 24 HIS providers. The inflection point indicates the optimal number of clusters based on the trade-off between complexity and variance.

Supplementary Material 15. Average silhouette score across different numbers of clusters (k), including the Cloud Offering variable

Note: The graph shows the quality of HIS provider clustering by how well each provider fits within its own cluster relative to other clusters. Scores closer to 1 indicate stronger separation, while scores close to zero indicate strongly overlapping clusters. Each number of clusters was tested using 50 random initializations to improve stability.

Supplementary Material 16. Clustering graph for k = 5 clusters based on HIS variables V1 to V5

Note: Results of k-means clustering are shown in a reduced two-dimensional space. Axes correspond to weighted combinations of HIS features that best summarize the information in two-dimensional space. Each point represents a single HIS provider, colored by membership to the respective cluster. Lines are drawn around the provider clusters to illustrate boundaries. Large shapes within each cluster show centers.

Supplementary Material 17. Overview of cluster centroids per HIS variable and descriptions for the alternative clustering approach, including Cloud Offering (V5)

|  | **Cluster centroids** | | | | |  |
| --- | --- | --- | --- | --- | --- | --- |
|  | $V_{1,p}$ | $V_{2,p}$ | $V_{3,p}$ | $V_{4,p}$ | $V_{5,p}$ |  |
| **Cluster** | Module  Utilization | Integration  Ratio | External Provider Variation | Maximum Hospital Size Coverage | Cloud  Offering | **Jaccard index** |
| Cluster A | 0.76 | 0.79 | 43.83 | 1,594 | 0 | 0.70 |
| Cluster B | 0.92 | 0.69 | 55.33 | 1,741 | 1 | 0.59 |
| Cluster C | 0.10 | 0.08 | 0.25 | 69 | 0 | 0.88 |
| Cluster D | 0.24 | 0.71 | 5.38 | 282 | 0 | 0.83 |
| Cluster E | 0.18 | 0.61 | 7.33 | 166 | 1 | 0.81 |

Note: Each row summarizes one of the five identified HIS provider clusters. To identify key characteristics, cluster centroids within each HIS variable column can be compared.

Supplementary Material 18. Provider cluster transitions between base clustering (k = 4) and alternative clustering with the Cloud Offering variable (k = 5)

Note: The diagram illustrates the flow of HIS providers between clusters when moving from clustering without the Cloud Offering variable to the clustering solution with the Cloud Offering variable. The width of the bands represents the number of providers transitioning. IDs indicate the specific providers that entered the newly formed cluster in the k = 5 solution. Small black numbers in flows indicate the cluster number.

Supplementary Material 19. Descriptive statistics of identified HIS provider clusters under an alternative clustering scenario with the Cloud Offering variable (k = 5)

|  |  | HIS provider cluster | | | |  |
| --- | --- | --- | --- | --- | --- | --- |
|  | Total  (N = 1,607) | Cluster A  (N = 700) | Cluster B  (N = 818) | Cluster C  (N = 7) | Cluster D  (N = 56) | Cluster E  (N = 26) |
| **DR-score** |  |  |  |  |  |  |
| Mean (SD) | 38.24 (10.83) | 38.73 (11.15) | 38.96 (9.98) | 21.76 (8.14) | 29.67 (9.43) | 24.83 (11.46) |
| **DR-score dimension** – Mean (SD) |  |  |  |  |  |  |
| Structures & Systems | 55.71 (17.26) | 57.59 (17.21) | 56.25 (15.97) | 24.53 (10.8) | 38.38 (16.45) | 33.49 (18.33) |
| Resilience Mgmt. & Performance | 45.38 (15.76) | 45.7 (15.97) | 45.64 (15.6) | 40.32 (11.85) | 42.56 (15.38) | 35.61 (13.51) |
| Organizational Control & Data Mgmt. | 40.64 (13.31) | 40.95 (13.89) | 41.43 (12.51) | 20.88 (11.11) | 34.18 (11.71) | 26.97 (10.72) |
| Clinical Processes | 38.76 (13.93) | 38.50 (14.29) | 40.57 (12.63) | 15.09 (8.75) | 26.00 (13.69) | 22.44 (14.6) |
| Information Exchange | 25.18 (10.15) | 25.73 (10.34) | 25.54 (9.75) | 12.92 (8.52) | 18.87 (8.94) | 16.08 (9.88) |
| Telehealth | 18.1 (14.03) | 19.20 (15.18) | 17.85 (12.75) | 15.34 (17.74) | 13.74 (14.68) | 6.49 (11.11) |
| Patient Participation | 5.25 (8.95) | 6.44 (11.22) | 4.40 (6.48) | 6.56 (14.78) | 2.55 (4.28) | 5.43 (8.97) |
| **DR workflow scores** – Mean (SD) |  |  |  |  |  |  |
| Administrative | 43.96 (13.49) | 44.23 (13.72) | 44.58 (13.18) | 31.38 (9.03) | 38.86 (12.00) | 31.48 (11.62) |
| Admission | 22.27 (11.63) | 23.03 (11.91) | 22.56 (10.98) | 16.07 (15.05) | 15.09 (11.97) | 10.31 (10.56) |
| Inpatient Treatment | 34.17 (12.29) | 34.03 (12.67) | 35.6 (11.21) | 13.86 (7.02) | 23.69 (11.73) | 20.92 (13.55) |
| Discharge | 11.31 (10.02) | 13.13 (11.5) | 10.11 (8.33) | 8.05 (13.18) | 8.00 (7.20) | 8.20 (11.99) |
| **Hospital type** (%) |  |  |  |  |  |  |
| Teaching | 878 (54.64) | 396 (56.57) | 466 (56.97) | 1 (14.29) | 13 (23.21) | 2 (7.69) |
| University | 54 (3.36) | 29 (4.14) | 24 (2.93) | 0 (0.00) | 1 (1.79) | 0 (0.00) |
| Psychiatric | 251 (15.62) | 93 (13.29) | 128 (15.65) | 5 (71.43) | 21 (37.50) | 4 (15.38) |
| **Ownership status** (%) |  |  |  |  |  |  |
| Public | 604 (37.58) | 227 (32.43) | 345 (42.18) | 3 (42.86) | 24 (42.86) | 5 (19.23) |
| Private non-profit | 456 (28.38) | 222 (31.71) | 188 (22.98) | 3 (42.86) | 25 (44.64) | 18 (69.23) |
| Private for-profit | 547 (34.04) | 251 (35.86) | 285 (34.84) | 1 (14.29) | 7 (12.50) | 3 (11.54) |
| **Number of beds** |  |  |  |  |  |  |
| Mean (SD) | 311.28 (315.77) | 343.12 (358.51) | 307.11 (280.56) | 48.86 (59.75) | 119.43 (136.13) | 69.23 (78.71) |
| < 250 beds (%) | 903 (56.20) | 379 (54.14) | 444 (54.28) | 7 (100.00) | 48 (85.71) | 25 (96.15) |
| 250 to 500 beds (%) | 416 (25.90) | 167 (23.86) | 241 (29.46) | 0 (0.00) | 7 (12.50) | 1 (3.85) |
| 501 to 700 beds (%) | 141 (8.80) | 68 (9.71) | 72 (8.80) | 0 (0.00) | 1 (1.79) | 0 (0.00) |
| > 700 beds (%) | 147 (9.10) | 86 (12.29) | 61 (7.46) | 0 (0.00) | 0 (0.00) | 0 (0.00) |
| **IT FTE per hospital bed** |  |  |  |  |  |  |
| Mean (SD) | 0.02 (0.03) | 0.02 (0.03) | 0.02 (0.03) | 0.05 (0.03) | 0.03 (0.04) | 0.05 (0.07) |
| **HIS provider module share** |  |  |  |  |  |  |
| Mean (SD) | 0.52 (0.26) | 0.41 (0.22) | 0.63 (0.22) | 0.38 (0.49) | 0.39 (0.35) | 0.29 (0.34) |
| **Federal state** (%) |  |  |  |  |  |  |
| Baden-Württemberg | 167 (10.39) | 65 (9.29) | 90 (11.00) | 0 (0.00) | 8 (14.29) | 4 (15.38) |
| Bavaria | 308 (19.17) | 157 (22.43) | 118 (14.43) | 1 (14.29) | 23 (41.07) | 9 (34.62) |
| Berlin | 54 (3.36) | 17 (2.43) | 34 (4.16) | 0 (0.00) | 1 (1.79) | 2 (7.69) |
| Brandenburg | 54 (3.36) | 25 (3.57) | 28 (3.42) | 0 (0.00) | 0 (0.00) | 1 (3.85) |
| Bremen | 12 (0.75) | 6 (0.86) | 6 (0.73) | 0 (0.00) | 0 (0.00) | 0 (0.00) |
| Hamburg | 33 (2.05) | 19 (2.71) | 8 (0.98) | 0 (0.00) | 1 (1.79) | 5 (19.23) |
| Hesse | 124 (7.72) | 45 (6.43) | 77 (9.41) | 1 (14.29) | 1 (1.79) | 0 (0.00) |
| Lower Saxony | 36 (2.24) | 15 (2.14) | 21 (2.57) | 0 (0.00) | 0 (0.00) | 0 (0.00) |
| Mecklenburg-Western Pomerania | 160 (9.96) | 60 (8.57) | 93 (11.37) | 1 (14.29) | 4 (7.14) | 2 (7.69) |
| North Rhine-Westphalia | 324 (20.16) | 150 (21.43) | 166 (20.29) | 0 (0.00) | 8 (14.29) | 0 (0.00) |
| Rhineland-Palatinate | 82 (5.10) | 35 (5.00) | 45 (5.50) | 0 (0.00) | 2 (3.57) | 0 (0.00) |
| Saarland | 19 (1.18) | 10 (1.43) | 8 (0.98) | 1 (14.29) | 0 (0.00) | 0 (0.00) |
| Saxony | 79 (4.92) | 34 (4.86) | 42 (5.13) | 0 (0.00) | 2 (3.57) | 1 (3.85) |
| Saxony-Anhalt | 49 (3.05) | 23 (3.29) | 25 (3.06) | 0 (0.00) | 1 (1.79) | 0 (0.00) |
| Schleswig-Holstein | 64 (3.98) | 23 (3.29) | 31 (3.79) | 3 (42.86) | 5 (8.93) | 2 (7.69) |
| Thuringia | 42 (2.61) | 16 (2.29) | 26 (3.18) | 0 (0.00) | 0 (0.00) | 0 (0.00) |
| **Hospital chain (%)^1^** |  |  |  |  |  |  |
| Chain-affiliated | 987 (61.42) | 438 (62.57) | 516 (63.08) | 2 (28.57) | 27 (48.21) | 4 (15.38) |
| Individual hospitals | 620 (38.58) | 262 (37.43) | 302 (36.92) | 5 (71.43) | 29 (51.79) | 22 (84.62) |

Note: Values are mean (SD) for continuous variables and frequency (%) for categorical variables. The total column summarizes overall hospital characteristics across all five HIS provider clusters. Percentages refer to the proportion of hospitals within each HIS provider cluster. 1. For descriptive analyses, hospital chain affiliation is reported as a binary variable (chain-affiliated vs. individual hospital). In the regression analyses, hospital chain is included as a categorical variable.

Supplementary Material 20. Ordinary least squares regression with HIS features as predictors under an alternative clustering scenario with the Cloud Offering variable

| **Dependent variables** | **Total** | **DR dimensional scores** | | | | | | | **DR workflow scores** | | | |
| --- | --- | --- | --- | --- | --- | --- | --- | --- | --- | --- | --- | --- |
|  | DR- score | Structures  & Systems | Resilience | Organizati-onal Control | Clinical  Processes | Information Exchange | Telehealth | SQRT(Patient Participation) | Admin-istrative | Admission | Inpatient Treatment | SQRT (Discharge) |
| **Model** | (1) | (D1) | (D2) | (D3) | (D4) | (D5) | (D6) | (D7) | (W1) | (W2) | (W3) | (W4) |
|  | Coefficient  (robust SE) | Coefficient  (robust SE) | Coefficient  (robust SE) | Coefficient  (robust SE) | Coefficient  (robust SE) | Coefficient  (robust SE) | Coefficient  (robust SE) | Coefficient  (robust SE) | Coefficient  (robust SE) | Coefficient  (robust SE) | Coefficient  (robust SE) | Coefficient  (robust SE) |
| **Predictors** |  |  |  |  |  |  |  |  |  |  |  |  |
| HIS features |  |  |  |  |  |  |  |  |  |  |  |  |
| Module Utilization | 6.882** (2.983) | 12.633***^†^ (4.592) | 8.683**^†^ (4.324) | 8.229**^†^ (3.667) | 7.308*  (4.179) | 2.809 (2.895) | -4.928 (3.885) | -0.018  (0.436) | 7.946**^†^ (3.606) | -1.614 (3.419) | 7.234**^†^ (3.606) | -0.315 (0.442) |
| Integration Ratio | 14.261*** (4.058) | 27.110***^†††^ (5.998) | 9.251* (5.276) | 13.261**^†^  (6.000) | 19.849***^†††^ (6.122) | 7.497* (4.022) | 1.483 (5.761) | -0.070  (0.689) | 11.848**^†^ (5.051) | 4.547 (5.093) | 17.673***^†††^ (5.112) | 0.136 (0.803) |
| Ext. Provider Variation | 0.001 (0.012) | -0.007  (0.018) | -0.016 (0.019) | -0.004 (0.014) | 0.018  (0.016) | 0.006 (0.012) | 0.018 (0.015) | -0.001  (0.002) | -0.009 (0.015) | 0.014 (0.013) | 0.013  (0.014) | 0.002 (0.002) |
| Max. Hospital Size | -0.001 (0.001) | -0.002  (0.001) | -0.001 (0.001) | -0.001 (0.001) | -0.001  (0.001) | 0.001 (0.001) | 0.002* (0.001) | 0.000  (0.000) | -0.001 (0.001) | 0.002 (0.001) | -0.001  (0.001) | 0.000 (0.000) |
| Cloud offering | -0.030 (0.823) | -0.793  (1.272) | -0.687 (1.260) | 0.380  (1.065) | 0.967  (1.150) | 0.244 (0.806) | -0.969 (0.957) | -0.047  (0.126) | -0.024 (1.051) | -0.420 (0.874) | 0.626  (1.004) | -0.065 (0.124) |
| Control variables |  |  |  |  |  |  |  |  |  |  |  |  |
| Teaching | 3.217*** (0.522) | 6.752*** (0.876) | 1.435* (0.823) | 2.042*** (0.674) | 4.584*** (0.744) | 1.674*** (0.551) | 4.477*** (0.749) | 0.125  (0.083) | 2.032*** (0.669) | 4.403*** (0.592) | 3.609*** (0.655) | 0.178** (0.082) |
| University | -0.595 (1.487) | -2.983  (2.056) | 1.337 (2.336) | 2.122  (1.841) | -3.274  (2.180) | 0.553 (1.567) | 2.780  (2.262) | 0.397  (0.289) | 1.336 (1.893) | 0.168 (1.636) | -1.323  (1.975) | -0.237 (0.207) |
| Psychiatric | 2.146*** (0.781) | 4.497*** (1.280) | 4.970*** (1.076) | 2.467** (1.074) | 1.469  (1.141) | 0.496 (0.773) | 0.754 (1.157) | 0.038  (0.110) | 3.803*** (0.918) | -1.100 (0.931) | 1.540  (1.011) | -0.268** (0.113) |
| *Ownership base: Public* |  |  |  |  |  |  |  |  |  |  |  |  |
| Ownership  (private non-profit) | -1.451 (1.136) | -5.347*** (1.763) | 1.486 (1.735) | -0.551 (1.393) | -2.979* (1.562) | -0.313 (1.125) | -1.904 (1.325) | 0.129  (0.155) | 0.142 (1.442) | -1.834 (1.131) | -1.898  (1.380) | 0.177 (0.157) |
| Ownership  (private for-profit) | 1.780** (0.906) | 3.813*** (1.426) | -0.843 (1.434) | 0.300  (1.156) | 3.416*** (1.245) | 1.211 (0.985) | 5.000*** (1.166) | -0.116  (0.128) | -0.568 (1.164) | 4.029*** (0.957) | 2.897*** (1.099) | 0.099 (0.136) |
| Number of beds | 0.008*** (0.001) | 0.017*** (0.002) | 0.004*** (0.002) | 0.006*** (0.001) | 0.010*** (0.001) | 0.004*** (0.001) | 0.005*** (0.001) | 0.001*** (0.000) | 0.005*** (0.001) | 0.007*** (0.001) | 0.009*** (0.001) | 0.001*** (0.000) |
| IT FTE per bed (log) | 5.835*** (1.278) | 8.157*** (2.021) | 8.650*** (1.909) | 5.815*** (1.544) | 5.509*** (1.765) | 2.521** (1.282) | 3.473** (1.665) | 0.335*  (0.193) | 7.228*** (1.592) | 1.788 (1.408) | 5.248*** (1.569) | 0.146 (0.195) |
| HIS provider module share | 4.333*** (1.356) | 6.466*** (2.119) | 3.635* (1.961) | 3.788** (1.698) | 6.889*** (1.885) | 0.176  (1.240) | 0.702 (1.576) | -0.115  (0.182) | 3.238** (1.649) | 0.959 (1.363) | 5.813*** (1.656) | -0.009 (0.192) |
| Federal state | Yes | Yes | Yes | Yes | Yes | Yes | Yes | Yes | Yes | Yes | Yes | Yes |
| Hospital chain | Yes | Yes | Yes | Yes | Yes | Yes | Yes | Yes | Yes | Yes | Yes | Yes |

Note: N = 1,607. Robust standard errors are shown in parentheses. Numbers smaller than 0.001 are displayed as 0.000. Significance is reported with raw p-values for the primary outcome (DR-score total) and with both raw p-values and Benjamini-Hochberg adjusted q-values within each predictor family for secondary outcomes (DR-score dimensions D1–D7 and workflow scores W1–W4). Significance for control variables is reported with raw p-values only. The following significance levels apply: *** p < 0.01, ** p < 0.05, * p < 0.10; ^†††^ q < 0.01, ^††^ q < 0.05, ^†^ q < 0.10.

Supplementary Material 21. Ordinary least squares regression with HIS clusters as predictors under an alternative clustering scenario with the Cloud Offering variable

| **Dependent variables** | **Total** | **DR dimensional scores** | | | | | | | **DR workflow scores** | | | |
| --- | --- | --- | --- | --- | --- | --- | --- | --- | --- | --- | --- | --- |
|  | DR- score | Structures  & Systems | Resilience | Organizati-onal Control | Clinical  Processes | Information Exchange | Telehealth | SQRT(Patient Participation) | Admin-istrative | Admission | Inpatient Treatment | SQRT (Discharge) |
| **Model** | (1) | (D1) | (D2) | (D3) | (D4) | (D5) | (D6) | (D7) | (W1) | (W2) | (W3) | (W4) |
|  | Coefficient  (robust SE) | Coefficient  (robust SE) | Coefficient  (robust SE) | Coefficient  (robust SE) | Coefficient  (robust SE) | Coefficient  (robust SE) | Coefficient  (robust SE) | Coefficient  (robust SE) | Coefficient  (robust SE) | Coefficient  (robust SE) | Coefficient  (robust SE) | Coefficient  (robust SE) |
| **Predictors** |  |  |  |  |  |  |  |  |  |  |  |  |
| HIS clusters |  |  |  |  |  |  |  |  |  |  |  |  |
| Base: Cluster A |  |  |  |  |  |  |  |  |  |  |  |  |
| Cluster B | -0.172 (0.702) | -1.852  (1.130) | -0.495 (1.109) | 0.498  (0.885) | 0.691  (0.976) | 0.441 (0.705) | -0.480 (0.892) | -0.108  (0.110) | 0.095  (0.894) | -0.184 (0.747) | 0.349  (0.860) | -0.049 (0.101) |
| Cluster C | -10.523*** (2.957) | -18.632***^†††^ (3.893) | -8.729***^††^ (3.317) | -11.092**^††^ (4.614) | -13.658***^†††^ (3.847) | -7.538**^†^ (3.542) | -2.465 (3.766) | 0.588  (0.863) | -10.489***^††^ (3.581) | -2.263 (5.495) | -12.264***^†††^ (3.065) | 0.011 (0.775) |
| Cluster D | -4.811*** (1.649) | -10.023***^†††^ (2.555) | -3.341 (2.679) | -4.900**^†^ (2.260) | -6.663***^††^ (2.285) | -2.776 (1.723) | 1.833  (2.420) | -0.325  (0.216) | -3.850*  (2.321) | -1.891 (1.895) | -5.545***^††^ (2.002) | -0.243 (0.242) |
| Cluster E | -7.702*** (2.608) | -10.607***^††^ (3.892) | -8.348**^††^ (3.409) | -8.269***^††^ (2.672) | -8.673***^††^ (3.259) | -4.371* (2.382) | -4.461* (2.417) | 0.184  (0.341) | -8.426***^††^ (2.914) | -4.401* (2.428) | -7.555**^††^ (3.034) | -0.297 (0.365) |
| Control variables |  |  |  |  |  |  |  |  |  |  |  |  |
| Teaching | 3.131*** (0.529) | 6.659*** (0.879) | 1.245 (0.828) | 1.905*** (0.675) | 4.558*** (0.753) | 1.645*** (0.553) | 4.487*** (0.750) | 0.125  (0.083) | 1.870*** (0.674) | 4.404*** (0.599) | 3.580*** (0.664) | 0.181** (0.083) |
| University | -0.555 (1.495) | -2.775  (2.065) | 1.445 (2.335) | 2.239  (1.840) | -3.391  (2.187) | 0.653 (1.567) | 2.765 (2.267) | 0.427  (0.286) | 1.444  (1.889) | 0.278 (1.645) | -1.447  (1.988) | -0.226 (0.208) |
| Psychiatric | 2.030*** (0.766) | 4.411*** (1.272) | 5.116*** (1.064) | 2.360** (1.060) | 1.243  (1.123) | 0.191 (0.774) | 0.216 (1.129) | 0.048  (0.108) | 3.792*** (0.901) | -1.560* (0.938) | 1.391  (0.997) | -0.302*** (0.113) |
| *Ownership base: Public* |  |  |  |  |  |  |  |  |  |  |  |  |
| Ownership  (private non-profit) | -1.187 (1.148) | -5.000*** (1.769) | 1.883 (1.748) | -0.238 (1.396) | -2.680* (1.571) | -0.269 (1.147) | -2.017 (1.320) | 0.126  (0.155) | 0.490  (1.452) | -1.876* (1.141) | -1.631  (1.392) | 0.173 (0.158) |
| Ownership  (private for-profit) | 1.771* (0.904) | 3.776*** (1.417) | -0.811 (1.445) | 0.333  (1.153) | 3.343*** (1.241) | 1.265 (0.979) | 5.062*** (1.165) | -0.122  (0.127) | -0.534  (1.170) | 4.052*** (0.949) | 2.841*** (1.095) | 0.096 (0.135) |
| Number of beds | 0.008*** (0.001) | 0.017*** (0.002) | 0.004*** (0.002) | 0.006*** (0.001) | 0.010*** (0.001) | 0.004*** (0.001) | 0.005*** (0.001) | 0.001*** (0.000) | 0.005*** (0.001) | 0.007*** (0.001) | 0.009*** (0.001) | 0.001***  (0.000) |
| IT FTE per bed (log) | 6.095*** (1.275) | 8.508*** (2.018) | 8.860*** (1.908) | 6.114*** (1.538) | 5.804*** (1.750) | 2.758** (1.288) | 3.788** (1.684) | 0.333*  (0.191) | 7.512*** (1.587) | 2.083 (1.423) | 5.469*** (1.561) | 0.169 (0.193) |
| HIS provider module share | 4.319*** (1.295) | 6.593*** (2.054) | 3.241* (1.887) | 3.683** (1.635) | 7.199*** (1.801) | 0.309 (1.203) | 0.460 (1.519) | -0.110  (0.181) | 2.918*  (1.575) | 1.005 (1.357) | 6.084*** (1.583) | 0.006 (0.184) |
| Federal state | Yes | Yes | Yes | Yes | Yes | Yes | Yes | Yes | Yes | Yes | Yes | Yes |
| Hospital chain | Yes | Yes | Yes | Yes | Yes | Yes | Yes | Yes | Yes | Yes | Yes | Yes |

Note: N = 1,607. Robust standard errors are shown in parentheses. Numbers smaller than 0.001 are displayed as 0.000. Significance is reported with raw p-values for the primary outcome (DR-score total) and with both raw p-values and Benjamini-Hochberg adjusted q-values within each predictor family for secondary outcomes (DR-score dimensions D1–D7 and workflow scores W1–W4). Significance for control variables is reported with raw p-values only. The following significance levels apply: *** p < 0.01, ** p < 0.05, * p < 0.10; ^†††^ q < 0.01, ^††^ q < 0.05, ^†^ q < 0.10.

**Supplementary Material 22.** Ordinary least squares regression results excluding the Structures and Systems dimension from overall DR-score

|  | **Dependent variable** | | **Comparison to primary OLS regression** | |
| --- | --- | --- | --- | --- |
|  | Total DR-score | Total DR-score | Change in direction | Magnitude of change |
|  | (1) | (2) |  |  |
| **Predictors** |  |  |  |  |
| HIS features |  |  |  |  |
| Module Utilization | 5.989** (2.898) |  | No | -0.882 |
| Integration Ratio | 12.026*** (3.788) |  | No | -2.322 |
| Ext. Provider Variation | 0.003 (0.012) |  | No | 0.002 |
| Max. Hospital Size | -0.001 (0.001) |  | No | 0.000 |
| HIS clusters |  |  |  |  |
| Base: Cluster A |  |  |  |  |
| Cluster B |  | 0.426 (0.885) | No | -0.198 |
| Cluster C |  | -9.338*** (3.101) | No | 1.022 |
| Cluster D |  | -5.185*** (1.478) | No | 0.550 |
| Control variables |  |  |  |  |
| Teaching | 2.581*** (0.511) | 2.563*** (0.512) | No/No | -0.636 / -0.635 |
| University | -0.150 (1.515) | -0.137 (1.526) | No/No | 0.444 / 0.448 |
| Psychiatric | 2.500*** (0.764) | 2.474*** (0.751) | No/No | 0.352 / 0.346 |
| Ownership base: Public |  |  |  |  |
| Ownership  (private non-profit) | -0.736 (1.103) | -0.539 (1.109) | No/No | 0.716 / -4.151 |
| Ownership  (private for-profit) | 1.415 (0.890) | 1.361 (0.886) | No/No | -0.365 / -0.350 |
| Number of beds | 0.006*** (0.001) | 0.006*** (0.001) | No/No | -0.002 / -0.002 |
| IT FTE per bed (log) | 5.514*** (1.240) | 5.670*** (1.234) | No/No | -0.320 / -0.338 |
| HIS provider module share | 4.020*** (1.301) | 4.141*** (1.212) | No/No | -0.304 / -0.236 |
| Federal state | Yes | Yes |  |  |
| Hospital chain | Yes | Yes |  |  |

Note: N = 1,607. Robust standard errors are shown in parentheses. This table reports OLS regression results excluding the *Structures and Systems* dimension score from the overall DR-score to assess potential overlap with HIS feature inputs (i.e., module availability and integration status). The right-hand columns compare results to the primary OLS model, indicating whether coefficient signs changed and the magnitude of change relative to the baseline model. Numbers smaller than 0.001 are displayed as 0.000. The following significance levels apply: *** p < 0.01, ** p < 0.05, * p < 0.10.
